# Interaction Between Treatment and Age or Sex in Non-ST-Segment Elevation Acute Coronary Disease and Three-Vessel Disease

**DOI:** 10.3389/fcvm.2022.879834

**Published:** 2022-06-02

**Authors:** Tianyu Li, Lin Jiang, Lianjun Xu, Jian Tian, Xueyan Zhao, Xinxing Feng, Dong Wang, Yin Zhang, Kai Sun, Jingjing Xu, Ru Liu, Bo Xu, Wei Zhao, Rutai Hui, Runlin Gao, Lei Song, Jinqing Yuan

**Affiliations:** ^1^National Clinical Research Center for Cardiovascular Disease, State Key Laboratory of Cardiovascular Disease, Fuwai Hospital, National Center for Cardiovascular Diseases, Chinese Academy of Medical Sciences and Peking Union Medical College, Beijing, China; ^2^Department of Cardiology, Fuwai Hospital, Chinese Academy of Medical Sciences and Peking Union Medical College, Beijing, China; ^3^Department of Endocrinology, Fuwai Hospital, Chinese Academy of Medical Sciences and Peking Union Medical College, Beijing, China; ^4^Information Center, Fuwai Hospital, Chinese Academy of Medical Sciences and Peking Union Medical College, Beijing, China

**Keywords:** non-ST-segment elevation acute coronary syndrome, three-vessel disease, coronary artery bypass grafting, percutaneous coronary intervention, age, sex

## Abstract

**Aims:**

To explore the effects of age and sex on the outcomes of coronary artery bypass grafting (CABG) and percutaneous coronary intervention (PCI) in non-ST-segment elevation acute coronary syndrome (NSTE-ACS) patients with the three-vessel disease (TVD).

**Methods and Results:**

The study is a subanalysis of data from a prospective cohort of 8,943 patients with angiographically confirmed TVD at Fuwai Hospital, Chinese Academy of Medical Sciences, Beijing, China. The primary end point was major adverse cardiac and cerebrovascular events (MACCEs), a composite of all-cause death, myocardial infarction, and stroke. In total, 2,819 patients with NSTE-ACS who received CABG (43.6%) or PCI (56.4%) were included, among whom 32.7% were of 65–74 years, 7.2% were ≥75 years, and 22.6% were women. The median follow-up duration was 6.8 years. The superiority of CABG relative to PCI in terms of MACCE was decreased with age (adjusted hazard ratio [HR] [95% confidence interval (CI)]: <65 years: 0.662 [0.495–0.885], *p* = 0.005; 65–74 years: 0.700 [0.512–0.956], *p* = 0.025; ≥75 years: 0.884 [0.529–1.479], *p* = 0.640) and was only seen in men (adjusted HR [95% CI]: men: 0.668 [0.526–0.848], *p* = 0.001; women: 0.713 [0.505–1.006], *p* = 0.054). Significant treatment-by-sex and treatment-by-age interactions were observed in patients ≥ 75 years and women, respectively, (*p*_interaction with sex_ = 0.001; *p*_interaction with age_ = 0.002).

**Conclusion:**

Coronary artery bypass grafting is favorable for most NSTE-ACS patients with TVD. The preponderance of CABG over PCI disappeared in patients ≥ 75 years and women. PCI is superior in women ≥ 75 years.

## Introduction

Myocardial revascularization, such as coronary artery bypass grafting (CABG) and percutaneous coronary intervention (PCI), plays an essential role in the management of coronary artery disease (CAD), especially for complex lesions, such as the three-vessel disease (TVD).

Age and sex are vital factors to consider when the heart team choosing revascularization strategies for patients with CAD and may be more critical for patients with non-ST-segment elevation acute coronary syndrome (NSTE-ACS), for the burden of NSTE-ACS is heavier in the elderly and women. The proportion of NSTE-ACS increases from 17% among CAD patients < 55 years to 56% among those ≥ 85 years, and remains above 50% among women regardless of age (1–3). However, a few studies have compared CABG and PCI, considering age and sex in the setting of NSTE-ACS. Due to the lack of evidence, current guidelines do not provide specific recommendations regarding the choice of revascularization strategies for this population (4, 5). We aimed to explore the effects of age and sex on outcomes of CABG and PCI in NSTE-ACS patients with TVD, providing evidence for real-world clinical decision-making.

## Materials and Methods

### Study Design, Setting, and Participants

The prospective cohort consisted of 8,943 consecutive patients with angiographically confirmed TVD from April 2004 to February 2011 at Fuwai Hospital, Chinese Academy of Medical Sciences, Beijing, China. Inclusion criteria were diagnosis of TVD and willingness to undergo follow-up. No prespecified exclusion criterion was applied. TVD was defined as stenosis of ≥50% in all three main epicardial coronary arteries (left anterior descending, left circumflex, and right coronary arteries), with or without the involvement of the left main coronary artery. Individual treatment strategy was determined through heart team discussion following contemporary practice guidelines (6, 7) and the patient’s preference (see Supplementary Methods). The study was complied with the Declaration of Helsinki. The Review Board of Fuwai Hospital approved the study protocol before enrollment [approval no. 2021–1579]. All participants provided written informed consent.

The last follow-up was finished in March 2016. Baseline and procedural data were collected into a database by independent clinical research coordinators. Outcome data were obtained through the telephone interviews, follow-up letters, or clinic visits. All events were carefully checked and verified by an independent group of clinical physicians. Investigator training, telephone recording, and blinded questionnaire filling were performed to achieve high-quality results.

### Present Analysis

The present study is a *post hoc* analysis of data from the cohort mentioned above to explore the effects of age and sex on the prognosis of CABG vs. PCI in NSTE-ACS patients with TVD. Patients with ST-segment elevation myocardial infarction (STEMI), stable CAD, and patients who received medical therapy only were excluded from the present analysis. NSTE-ACS included non-ST-segment elevation myocardial infarction (NSTEMI) and unstable angina and was diagnosed according to guidelines at the time (8). Age was categorized as <65 years, 65–74 years, and ≥75 years according to the recommendations of the American Heart Association Council on Clinical Cardiology and Society of Geriatric Cardiology (9).

### End Points

The primary end point was major adverse cardiac and cerebrovascular events (MACCEs), a composite of all-cause death, myocardial infarction (MI), and stroke. Secondary end points included the individual components of MACCE, cardiac death, and unplanned revascularization. All deaths were considered cardiac unless an unequivocal non-cardiac cause could be established. MI was defined by the consensus document from the Joint European Society of Cardiology/American College of Cardiology Committee for the redefinition of MI (10). Stroke was defined as self-reported history of ischemic and hemorrhagic stroke. Unplanned revascularization was defined as repeated CABG or PCI of any vessel for ischemic symptoms and events.

### Statistical Analysis

In the main analysis, inverse probability of treatment weighting (IPTW) based on propensity score was used to balance the observed covariates between groups (11). The propensity score was generated by a multivariable logistic regression model with 17 prognostically meaningful or confounding covariates (Table 1). Patients who underwent CABG were weighted by marginal probability of CABG/propensity score, and patients who underwent PCI were weighted by marginal probability of PCI/[1 – propensity score]. Baseline characteristics were compared between groups using standardized differences; the absolute value of ≥0.1 indicates a significant imbalance (12). Clinical events were compared between groups using Pearson’s chi-square test or Fisher’s exact test. Cumulative incidences of clinical events were calculated using the Kaplan–Meier method and were compared using the log-rank test. Cox proportional-hazards regression models were used to estimate hazard ratios (HRs) and 95% confidence intervals (CIs) of the CABG group when compared with the PCI group. Log-minus-log plots verified the proportional hazards assumption. Missing values were imputed with the median for continuous variables or the mode for categorical variables, except for the synergy between PCI with Taxus and Cardiac Surgery (SYNTAX) score. Two-tailed values of *p* < 0.05 were considered to be statistically significant.

**TABLE 1:** Baseline characteristics.

	Original cohort (*n* = 2,819)			Weighted cohort		
	PCI (*n* = 1,589)	CABG (*n* = 1,230)	Std. diff.	PCI	CABG	Std. diff
Demographic characteristics						
Sex^a^ (Women)	384 (24.2)	252 (20.5)	0.088	22.7	22.5	0.005
Age^a^, years	61 [54, 69]	62 [55, 69]	0.062	61.4 ± 10.0	61.3 ± 8.9	0.014
Age group						
<65 years	949 (59.7)	744 (60.5)	0.016	58.9	60.6	0.036
65–74 years	502 (31.6)	421 (34.2)	0.056	32.1	34.5	0.051
≥75 years	138 (8.7)	65 (5.3)	0.134	9.0	4.9	0.164
Clinical characteristics						
BMI, kg/m^2^	26.0 [24.0, 27.6]	25.9 [24.0, 27.6]	0.004	25.8 ± 3.0	25.9 ± 3.0	0.059
Smoking history^a^	861 (54.2)	660 (53.7)	0.011	53.2	53.2	0.001
Drinking history^a^	448 (28.2)	323 (26.3)	0.043	27.8	28.4	0.014
Comorbidities						
Diabetes^a^	535 (33.7)	415 (33.7)	0.001	34.9	31.9	0.064
Hypertension^a^	1,133 (71.3)	873 (71.0)	0.007	71.0	71.7	0.017
Dyslipidemia^a^	947 (59.6)	694 (56.4)	0.064	57.7	57.1	0.012
Peripheral artery disease^a^	61 (3.8)	152 (12.4)	0.316	9.1	7.5	0.059
COPD	17 (1.1)	14 (1.1)	0.009	1.3	1.0	0.027
Anemia	8 (0.5)	3 (0.2)	0.043	0.4	0.2	0.032
History						
Prior MI^a^	505 (31.8)	461 (37.5)	0.120	35.6	35.0	0.012
Prior stroke^a^	148 (9.3)	130 (10.6)	0.042	10.1	9.5	0.021
Prior PCI^a^	210 (13.2)	120 (9.8)	0.109	11.9	13.5	0.047
Prior CABG^a^	71 (4.5)	8 (0.7)	0.244	2.8	4.0	0.067
NSTEMI^a^	269 (17.0)	143 (11.6)	0.152	13.8	11.1	0.083
eGFR360.0 ml/min/1.73 m^2a^	115 (7.2)	126 (10.2)	0.107	11.3	11.1	0.007
LVEF < 40%^a^	23 (1.4)	42 (3.4)	0.128	2.9	1.9	0.068
Left main disease^a^	198 (12.5)	459 (37.3)	0.600	25.2	23.3	0.045
SYNTAX score^a^	21.0 [15.0, 27.0]	30.3 [24.0, 36.5]	0.848	24.7 ± 9.9	25.8 ± 12.6	0.102
0–22	923 (58.1)	248 (20.2)	0.842	40.8	42.0	0.023
23–32	514 (32.4)	505 (41.2)	0.184	35.4	35.9	0.012
≥33	151 (9.5)	473 (38.6)	0.723	23.8	22.1	0.040
Post-procedural medication						
Aspirin	1,558 (98.0)	1,153 (93.7)	0.218	97.6	95.3	0.125
Clopidogrel	1,476 (92.9)	98 (8.0)	3.217	89.7	14.3	2.848
Satins	1,412 (88.9)	202 (16.4)	2.108	88.9	17.9	2.024
Beta blockers	1,411 (88.8)	1,064 (86.5)	0.070	89.4	88.1	0.040
ACEIs/ARBs	1,056 (66.5)	161 (13.1)	1.301	63.9	14.0	1.189

*Values are expressed as number (%) or median [interquartile range] for the original cohort, and percent or mean ± standard deviation (SD) for the weighted cohort.*

*PCI, percutaneous coronary intervention; CABG, coronary artery bypass grafting; Std. diff., standardized difference; BMI, body mass index; COPD, chronic obstructive pulmonary disease; MI, myocardial infarction; NSTEMI, non-ST-segment elevation myocardial infarction; eGFR, estimated glomerular filtration rate; LVEF, Left ventricular ejection fraction; SYNTAX, synergy between percutaneous coronary intervention with Taxus and cardiac surgery; ACEI, angiotensin converting enzyme inhibitor; and ARB, angiotensin-receptor blocker.*

*^a^Candidate variable selected to generate propensity score.*

Subgroup analysis was performed to assess the primary end point according to five prespecified variables of interest: subtypes of NSTE-ACS (NSTEMI or unstable angina), presence of diabetes, baseline estimated glomerular filtration rate ≤ 60 vs. >60 ml/min/1.73 m^2^, left main disease, and preprocedure SYNTAX score.

*E*-value was applied to quantify the magnitude of unmeasured and residual confounding that could negate the observed results (13). Covariate adjustment using propensity score was applied as a sensitivity analysis to assess the robustness of our findings. All analyses were conducted with R version 3.6.3 (R Core Team (14), Vienna, Austria)^1^. Figures were created by GraphPad Prism version 8.0.2 (GraphPad Software, San Diego, CA, United States)^2^.

## Results

### Study Population and Baseline Characteristics

**FIGURE 1:**
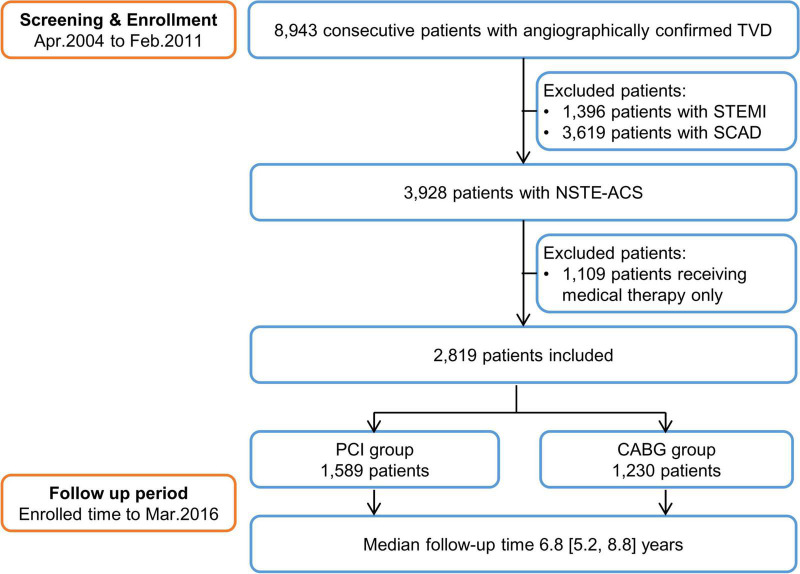
The study flow chart. TVD, three-vessel disease; STEMI, ST-segment elevation myocardial infarction; SCAD, stable coronary artery disease; NSTE-ACS, non-ST-segment elevation acute coronary syndrome; PCI, percutaneous coronary intervention; and CABG, coronary artery bypass grafting.

After excluding 1,396 patients with STEMI, 3,619 patients with CAD, and 1,109 patients received medical therapy only, the study population consisted of 2,819 NSTE-ACS patients who received CABG (43.6%) or PCI (56.4%). The median follow-up duration was 6.8 years (interquartile range: 5.2–8.8; Figure 1). The mean age of the study population was 61.3 years, 22.6% were women, and 14.6% presented with NSTEMI. The numbers of missing values and corresponding dispositions are shown in Supplementary Table 1.

Table 1 shows baseline characteristics of the study population before and after weighting. In the original cohort, the PCI group had more women, more patients ≥ 75 years, and more patients presenting with NSTEMI. Patients in the CABG group more often had peripheral artery disease, prior MI, or a left ventricular ejection fraction < 40%, but were less likely to have prior revascularization. Lesion characteristics were more complex in the CABG group, manifested as more left main disease and higher SYNTAX score. Four classes of secondary prevention drugs were less prescribed after CABG, especially statins and angiotensin-converting enzyme inhibitors/angiotensin-receptor blockers (ACEIs/ARBs). Baseline characteristics were well balanced after weighting except for a higher proportion of patients ≥ 75 years and a higher prescription rate of post-procedural medication in the PCI group.

Supplementary Tables 2, 3 show participants’ baseline characteristics by age category and sex. Older patients and women were more likely to be treated with PCI. Patients’ median age was significantly different between CABG and PCI groups in the age categories of <65 years and ≥75 years. The proportion of NSTEMI was increased with age, and women were more likely to present with NSTEMI than men. Regardless of age and sex, patients in the CABG group were more likely to have complex lesions but less likely to have prior revascularization and post-procedural medication.

### Outcomes for the Overall Study Population

**FIGURE 2:**
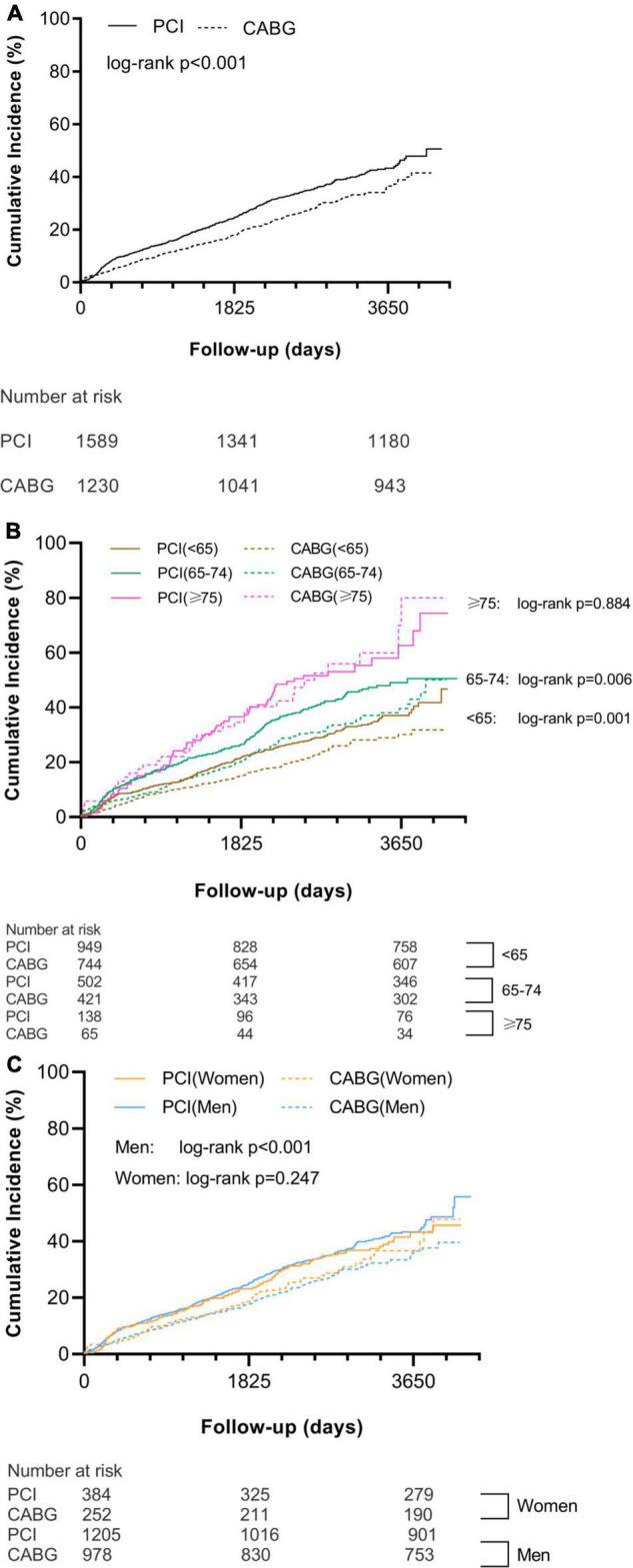
Cumulative incidence of MACCE in all patients **(A)**, different age categories **(B)**, and different sex groups **(C)**. MACCE, major adverse cardiovascular and cerebrovascular event; PCI, percutaneous coronary intervention; and CABG, coronary artery bypass grafting.

Major adverse cardiac and cerebrovascular events were occurred in 23.8% of patients in the CABG group and 26.4% of patients in the PCI group (adjusted HR [95% CI]: 0.676 [0.553–0.828], *p* < 0.001; Supplementary Table 4, Figure 2A, and Table 2), mainly due to reduced MI. Strokes were occurred in more patients after CABG than after PCI. The CABG group experienced a significantly lower incidence of unplanned revascularization. Cumulative incidences of all-cause death and cardiac death showed no difference between CABG and PCI groups. However, the risk of cardiac death was significantly lower in the CABG group than in the PCI group after adjustment (Supplementary Table 4, Supplementary Figures 1–5, and Table 2).

**TABLE 2:** IPTW-adjusted risks of CABG relative to PCI for clinical events.

		All patients (*n* = 2,819)		<65 years (*n* = 1,693)		65–74 years (*n* = 923)		≥75 years (*n* = 203)		*p* *
		Adjusted HR [95% CI]	*p*	Adjusted HR [95% CI]	*p*	Adjusted HR [95% CI]	*p*	Adjusted HR [95% CI]	*p*	
MACCE	All	0.676 [0.553–0.828]	<0.001	0.662 [0.495–0.885]	0.005	0.700 [0.512–0.956]	0.025	0.884 [0.529–1.479]	0.640	0.441
	Women	0.713 [0.505–1.006]	0.054	0.600 [0.336–1.072]	0.084	0.644 [0.404–1.028]	0.065	3.272 [1.421–7.531]	0.005	**0.002**
	Men	0.668 [0.526–0.848]	0.001	0.674 [0.486–0.933]	0.018	0.722 [0.492–1.060]	0.097	0.578 [0.332–1.005]	0.052	0.746
	*p* ^a^		0.409		0.697		0.643		**0.001**	
All-cause death	All	0.803 [0.566–1.138]	0.217	0.684 [0.388–1.206]	0.190	1.001 [0.615–1.630]	0.997	0.868 [0.467–1.611]	0.653	0.303
	Women	0.854 [0.526–1.388]	0.525	0.268 [0.093–0.772]	0.015	1.059 [0.574–1.952]	0.855	2.891 [1.016–8.227]	0.047	**0.003**
	Men	0.793 [0.518–1.212]	0.283	0.797 [0.412–1.541]	0.499	0.986 [0.533–1.824]	0.965	0.542 [0.286–1.028]	0.061	0.223
	*p* ^a^		0.383		0.056		0.879		**0.003**	
Myocardial infarction	All	0.295 [0.189–0.461]	<0.001	0.369 [0.216–0.629]	0.000	0.178 [0.072–0.439]	<0.001	0.272 [0.055–1.343]	0.110	0.237
	Women	0.419 [0.163–1.077]	0.071	0.936 [0.262–3.343]	0.919	0.210 [0.038–1.154]	0.073	0.924 [0.087–9.832]	0.948	0.218
	Men	0.266 [0.160–0.441]	<0.001	0.318 [0.175–0.576]	0.000	0.159 [0.063–0.404]	<0.001	0.156 [0.018–1.384]	0.095	0.440
	*p* ^a^		0.343		0.119		0.737		0.394	
Stroke	All	1.874 [1.299–2.701]	0.001	1.722 [1.070–2.771]	0.025	2.141 [1.193–3.843]	0.011	2.112 [0.754–5.922]	0.155	0.711
	Women	1.237 [0.693–2.208]	0.471	1.539 [0.589–4.021]	0.379	1.091 [0.504–2.360]	0.825	3.673 [0.567–23.779]	0.172	0.664
	Men	2.120 [1.371–3.279]	0.001	1.755 [1.032–2.986]	0.038	3.071 [1.432–6.584]	0.004	1.955 [0.633–6.037]	0.244	0.213
	*p* ^a^		0.116		0.781		**0.018**		0.821	
Cardiac death	All	0.544 [0.340–0.868]	0.011	0.509 [0.224–1.157]	0.107	0.598 [0.334–1.072]	0.084	0.828 [0.258–2.655]	0.751	0.621
	Women	0.559 [0.251–1.243]	0.154	0.115 [0.015–0.911]	0.041	0.657 [0.258–1.669]	0.377	2.077 [0.481–8.973]	0.327	**0.015**
	Men	0.543 [0.309–0.954]	0.034	0.611 [0.237–1.572]	0.306	0.579 [0.279–1.204]	0.143	0.354 [0.106–1.191]	0.094	0.716
	*p* ^a^		0.787		0.060		0.875		**0.031**	
Unplanned revasculari zation	All	0.230 [0.153–0.347]	<0.001	0.257 [0.156–0.422]	<0.001	0.187 [0.088–0.399]	<0.001	0.092 [0.011–0.733]	0.024	0.561
	Women	0.207 [0.078–0.547]	0.002	0.301 [0.093–0.980]	0.046	0.053 [0.007–0.403]	0.005	0.485 [0.051–4.620]	0.529	0.262
	Men	0.236 [0.150–0.371]	<0.001	0.248 [0.143–0.429]	<0.001	0.229 [0.101–0.518]	<0.001	–	–	0.383
	*p* ^a^		0.761		0.733		0.164		0.215	

*Interaction p with age.

*^a^Interaction p with sex.*

*IPTW, inverse probability of treatment weighting; CABG, coronary artery bypass grafting; PCI, percutaneous coronary intervention; HR, hazard ratio; CI, confidence interval; and MACCE, major adverse cardiac and cerebrovascular events. Bold indicates statistical significance.*

### Effect of Age on Outcomes of Coronary Artery Bypass Grafting vs. Percutaneous Coronary Intervention

**FIGURE 3:**
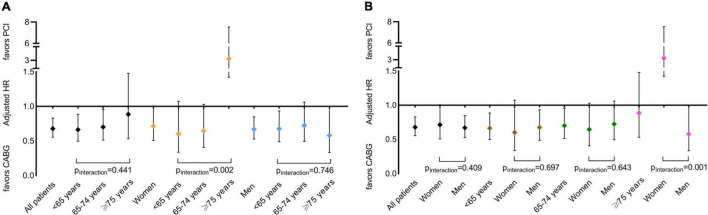
Effect of CABG relative to PCI on MACCE by age categories **(A)** and sex groups **(B)**. PCI, percutaneous coronary intervention; CABG, coronary artery bypass grafting; and MACCE, major adverse cardiovascular and cerebrovascular event.

The incidence of MACCE was increased with age regardless of revascularization strategy. The superiority of CABG relative to PCI tended to decrease with age. CABG significantly reduced MACCE when compared with PCI in the two younger age categories (<65 years: 18.5% vs. 20.9, adjusted HR [95% CI]: 0.662 [0.495–0.885], *p* = 0.005; 65–74 years: 29.2 vs. 31.3%, adjusted HR [95% CI]: 0.700 [0.512–0.956], *p* = 0.025). In patients ≥ 75 years, the risk for MACCE of CABG relative to PCI was neutral (49.2 vs. 46.4%, adjusted HR: 0.884 [0.529–1.479], *p* = 0.640; Supplementary Table 4, Figure 2B, and Table 2). Significant treatment-by-age interaction was observed in women (*p*_interaction_ = 0.002), as the risk for MACCE of CABG was comparable to those of PCI in the two younger age categories (<65 years: 17.6 vs. 17.9%, adjusted HR [95% CI]: 0.600 [0.336–1.072], *p* = 0.084; 65–74 years: 30.7 vs. 33.9%, adjusted HR [95% CI]: 0.644 [0.404–1.028], *p* = 0.065) but was significantly higher in patients ≥ 75 years (61.5 vs. 37.8%, adjusted HR [95% CI]: 3.272 [1.421–7.531], *p* = 0.005; Supplementary Table 4, Table 2, and Figure 3A).

For secondary end points, CABG was associated with a lower risk of MI and a higher risk of stroke than PCI in the two younger age categories, whereas the risks were neutral in patients ≥ 75 years. CABG was superior to PCI in unplanned revascularization and was comparable to PCI in all-cause death and cardiac death in all three age categories. Interactions between age category and revascularization strategy for secondary end points were not significant (*p*_*interaction*_ range: 0.237–0.926; Supplementary Table 4, Supplementary Figures 1–5, and Table 2).

### Effect of Sex on Outcomes of Coronary Artery Bypass Grafting vs. Percutaneous Coronary Intervention

Compared with PCI, CABG significantly reduced MACCE in men but not in women (men: 23.3 vs. 26.0%, adjusted HR [95% CI]: 0.668 [0.526–0.848], *p* = 0.001; women: 25.8 vs. 27.6%, adjusted HR [95% CI]: 0.713 [0.505–1.006], *p* = 0.054; Supplementary Table 4, Figure 2C, and Table 2). Significant treatment-by-sex interaction was observed in patients ≥ 75 years (*p*_interaction_ = 0.001), as CABG trended to be favored in men (46.2 vs. 50.5%, adjusted HR [95% CI]: 0.578 [0.332–1.005], *p* = 0.052), whereas PCI was significantly favored in women (61.5 vs. 37.8%, adjusted HR [95% CI]: 3.272 [1.421–7.531], *p* = 0.005; Supplementary Table 4, Table 2, and Figure 3B).

For secondary end points, the comparison of CABG and PCI in men demonstrated similar results in the entire cohort. While in women, only unplanned revascularization was significantly lower after CABG than PCI; other outcome measures between treatment groups showed no significant difference. No significant interaction between sex and revascularization strategy was found in the entire cohort and each age category, except that in women ≥ 75 years, the risk of all-cause death was three times higher after CABG than PCI with a significant treatment-by-sex interaction (*p*_interaction_ = 0.003; Supplementary Table 4, Supplementary Figures 1–5, and Table 2).

### Subgroup Analysis and Sensitivity Analysis

The risk of MACCE was significantly lower in patients who received CABG than in those who received PCI, regardless of the presence of diabetes. CABG achieved a significantly lower risk of MACCE than PCI in the subgroups of unstable angina, non-diabetes, left main uninvolved, and intermediate (23–32) to high (≥33) SYNTAX score, whereas a comparable risk to PCI in the complementary subgroups. No significant interaction between age or sex and revascularization strategy was observed in each subgroup (Supplementary Table 5 and Supplementary Figure 6).

The *E*-values for IPTW-adjusted HRs and CIs were greater than the association of all measured covariates with all outcomes except for cardiac death (Supplementary Tables 6, 7). It is unlikely that unmeasured and residual confounding would have a substantially stronger association with both revascularization strategy and MACCE than these known risk factors to negate the observed results. Sensitivity analysis yielded consistent results with the main findings (Supplementary Tables 5, 8).

## Discussion

Explicitly focused on NSTE-ACS patients with TVD, through a median of 6.8 years of follow-up, our study demonstrated that in real-world clinical practice, CABG had better outcomes in terms of MACCE, MI, cardiac death, and unplanned revascularization, but a higher risk of stroke; the benefit of CABG over PCI was not observed in patients ≥ 75 years or women, further analysis indicated that women ≥ 75 years had significantly higher risks of MACCE and all-cause death after CABG than PCI.

Our finding is aligned with the result of Chang et al.’s meta-analysis, i.e., NSTE-ACS patients with TVD of the SYNTAX, randomized comparison of coronary artery bypass surgery and everolimus-Eluting stent implantation in the treatment of patients with multivessel coronary artery disease (BEST), and premier of randomized comparison of bypass surgery versus angioplasty using sirolimus-eluting stent in patients with left main coronary artery disease (PRECOMBAT) trials, that CABG had a lower incidence of 5-year composite end point, mainly driven by a reduction in MI (14). We also found a lower cardiac death rate achieved by CABG, although the all-cause death rate was comparable. Two observational studies reported a significant 10-year survival benefit with CABG over PCI (15, 16), whereas other studies reported inconsistent results (17, 18). CABG significantly reduces repeated revascularization when compared with PCI and has been a universal finding across manifestations of CAD. The superiority of CABG might be attributable to better completeness and durability of revascularization. Strokes occurred more frequently after CABG than PCI (19). Possible mechanisms for the higher incidence of perioperative stroke in the CABG group include aortic manipulation, intraoperative hypoperfusion, early post-operative low cardiac output syndrome, post-operative atrial fibrillation, and hypercoagulability (in particular, preoperative interruption of antiplatelet therapy can lead to rebound hypercoagulation; 20). Routine dual antiplatelet therapy might explain the lower incidence of long-term stroke in the PCI group.

In the subgroup analysis, we only found that CABG was superior to PCI in patients without diabetes but not in diabetic patients. In addition, we found that the superiority of CABG over PCI was pronounced in patients without left main disease and patients with intermediate-to-high SYNTAX scores, which supports most existing evidence from randomized trials (14, 21, 22). Subtypes of NSTE-ACS did not affect the advantage of CABG relative to PCI in our study. These findings indicate that CABG is generally preferable among NSTE-ACS patients with TVD.

We noticed a trend that in NSTE-ACS patients with TVD, the advantage of CABG relative to PCI was decreased with age. In patients ≥ 75 years, clinical outcomes were not significantly different between treatment groups except for less unplanned revascularization after CABG. Similar results were obtained in subgroups. Analyses restricted to patients with NSTE-ACS yield consistent results with our findings (15). On the contrary, other studies consisting of heterogeneous populations reported a more striking benefit of CABG in the elderly than in younger patients (15, 23–25). Notably, patients with NSTE-ACS were only accounted for <10–30% of the participants in these studies. Evidence derived from these studies cannot be extended to the NSTE-ACS population. The difference may attribute to the different pathophysiology of NSTE-ACS. Atypical symptoms, non-diagnostic electrocardiographs, and lower specificity of high-sensitivity troponin are more common in elderly patients with NSTE-ACS, which contributes to delays in diagnosis and revascularization, offsetting the benefit of CABG (9, 26). Additionally, the categorization of age varies among studies, which might also cause conflicting results.

As for the analysis by sex, the advantage of CABG relative to PCI was prominent in men, while in women, CABG only reduced unplanned revascularization. This trend was consistent in each subgroup. Other studies reported similar results but controversial treatment-by-sex interaction (14, 16, 22). Possible reasons for the lessened advantage of CABG in women are as follows. First, advanced age, comorbidities, and atypical symptoms that are more common in women with CAD mean higher surgical risk (27, 28). Second, women are more likely to have non-obstructive CAD, signifying different pathophysiology, such as microvascular disease and endothelial dysfunction, for which revascularization has limited effectiveness (27). Third, for women, smaller vessels increase the difficulties of surgical revascularization; less use of the internal mammary artery decreases the long-term patency of grafts (1).

We further revealed that women ≥ 75 years suffered from a significantly higher risk of MACCE after CABG than PCI with significant treatment-by-sex and treatment-by-age interactions, primarily driven by the higher risk of all-cause death. NSTE-ACS, advanced age, and women may characterize a population at increased risk of CABG. It is commonly observed that patients with NSTE-ACS are more likely to be elderly and women, and women account for over half of patients with ACS ≥ 75 years (1). Besides, major modifiable cardiovascular risk factors are substantially less presented in young women than in age-matched men, but women ≥ 75 years have a greater incidence of these factors as compared to age-matched men. The impact of these factors on cardiovascular risk and arterial stiffness is more potent in women as compared to men (29, 30). However, given that there were only 58 women ≥ 75 years with 25 primary end point events, the possibility that our finding was observed by chance cannot be ruled out. This finding should be considered as a hypothesis and needs further confirmation.

The study has some limitations. First, patients were not randomized to each treatment group. Second, small sample sizes and the small number of events in the age category of ≥ 75 years, women, and some subgroups limited the power to detect statistical significance. Third, procedural characteristics were not collected in the study, such as vascular access, type of stent or vessel graft, and completeness of revascularization; thus, the study could not describe the changes in procedural techniques during the recruitment period. Forth, secondary prevention drugs were underused after CABG, especially statins and ACEIs/ARBs. Even after IPTW adjustment, this deficiency still existed. Finally, this single-center study was conducted at an urban tertiary cardiovascular hospital, limiting the generalization of our findings.

In summary, our findings support that CABG is favorable for most NSTE-ACS patients with TVD. Taking age and sex into consideration, the less invasive PCI can be an alternative to CABG in patients ≥ 75 years and women because of its non-inferior outcomes and faster and more comfortable post-procedural recovery. Particularly, PCI is superior to CABG in women ≥ 75 years.

## Data Availability Statement

The raw data supporting the conclusions of this article will be made available by the authors, without undue reservation.

## Ethics Statement

The studies involving human participants were reviewed and approved by Review Board of Fuwai Hospital. The patients/participants provided their written informed consent to participate in this study.

## Author Contributions

TL: conceptualization, methodology, formal analysis, and writing – original draft. LJ: formal analysis and writing – review and editing. LX: validation. JT, XF, DW, YZ, JX, and RL: data curation. XZ: validation and writing – review and editing. KS: software, resources, and visualization. BX: investigation and resources. WZ: software and resources. RH and RG: supervision. LS: conceptualization, investigation, writing – review and editing, supervision, and funding acquisition; JY: conceptualization, investigation, supervision, project administration, and funding acquisition. All authors contributed to the article and approved the submitted version.

## Conflict of Interest

The authors declare that the research was conducted in the absence of any commercial or financial relationships that could be construed as a potential conflict of interest.

## Publisher’s Note

All claims expressed in this article are solely those of the authors and do not necessarily represent those of their affiliated organizations, or those of the publisher, the editors and the reviewers. Any product that may be evaluated in this article, or claim that may be made by its manufacturer, is not guaranteed or endorsed by the publisher.

## Footnotes

1
www.R-project.org

2
www.graphpad.com
