# Supplementary material for: Interaction Between Treatment and Age or Sex in Non-ST-Segment Elevation Acute Coronary Disease and Three-Vessel Disease
Source: Front Cardiovasc Med. 2022 Jun 2;9:879834. doi: 10.3389/fcvm.2022.879834 (PMC9200996; doi:10.3389/fcvm.2022.879834)
Supplement: Supplementary file 1 [file Table_1.DOCX]

Supplementary Material

# Contents

[Supplementary Methods](#SP_M)

[Supplementary Table 1 Numbers of missing values and corresponding dispositions. 3](#_Toc81960342)

[Supplementary Table 2 Baseline characteristics according to age category. 4](#_Toc81960343)

[Supplementary Table 3 Baseline characteristics according to sex. 6](#_Toc81960344)

[Supplementary Table 4 Cumulative incidences of clinical events: PCI versus CABG. 7](#_Toc81960345)

[Supplementary Table 5 Subgroup analysis of MACCE in special population. 8](#_Toc81960346)

[Supplementary Table 6 E-value for IPTW adjusted hazard ratio and limit of the confidence interval closest to the null. 10](#_Toc81960347)

[Supplementary Table 7 The largest covariate-outcome associations for each outcome. 11](#_Toc81960348)

[Supplementary Table 8 PS adjusted risks of CABG relative to PCI for clinical events. 12](#_Toc81960349)

[Supplementary Figure 1 Cumulative incidence of all-cause death. 13](#_Toc82813559)

[Supplementary Figure 2 Cumulative incidence of myocardial infarction. 14](#_Toc82813560)

[Supplementary Figure 3 Cumulative incidence of stroke. 15](#_Toc82813561)

[Supplementary Figure 4 Cumulative incidence of cardiac death. 16](#_Toc82813562)

[Supplementary Figure 5 Cumulative incidence of unplanned revascularization. 17](#_Toc82813563)

[Supplementary Figure 6 IPTW adjusted hazard ratio of CABG relative to PCI for MACCE stratified by age group and sex in subgroups. 18](#_Toc82813564)

# Supplementary Methods

Our heart team typically consists of interventional cardiologists, cardiac surgeons and other physicians from department of cardiology.

Patients with suspected NSTE-ACS underwent invasive coronary angiography in our Cath lab. Depending on the results of coronary angiography, a patient might be referred for medical therapy, ad-hoc PCI, selective PCI or surgical CABG after a heart-team discussion

Typical characteristics favoring PCI include low SYNTAX score (0-22), severe comorbidity and poor general condition (reduced life expectancy, frailty, etc.). Typical characteristics favoring CABG include higher SYNTAX score (over 32), diabetes, reduced LVEF, contraindication to DAPT, recurrent in-stent restenosis, etc. Medical therapy is assigned to patients not in need of revascularization therapy (i.e. stenosis caused by stable plaque ≥50% stenotic yet not severe enough for revascularization) or patients who are clinically unsuitable for either revascularization procedure (CABG or PCI).

Patients who refused to receive the assigned treatment chose an alternative strategy based on heart team suggestions and their own preferences

# Supplementary Tables

**Supplementary Table 1 Numbers of missing values and corresponding dispositions.**

| **Variables** | **Number of missing values** | **Disposition** |
| --- | --- | --- |
| BMI | 22 (0.8%) | Median imputation |
| Prior stroke | 5 (0.2%) | Mode imputation |
| Dyslipidemia | 3 (0.1%) | Mode imputation |
| Smoking history | 1 (0) | Mode imputation |
| LVEF | 7(1.2%) | Median imputation |
| SYNTAX score | 5 (0.2%) | Undisposed |

BMI, body mass index; LVEF, left ventricular ejection fraction; SYNTAX, synergy between percutaneous coronary intervention with taxus and cardiac surgery.

**Supplementary Table 2 Baseline characteristics according to age category.**

|  | **<65 years (n=1,693)** | | | **65-74 years (n=923)** | | | **≥75 years (n=203)** | | |
| --- | --- | --- | --- | --- | --- | --- | --- | --- | --- |
|  | **PCI (n=949)** | **CABG (n=744)** | **p** | **PCI (n=502)** | **CABG (n=421)** | **p** | **PCI (n=138)** | **CABG (n=65)** | **p** |
| **Demographic characteristics** |  |  |  |  |  |  |  |  |  |
| Sex (Women) | 162 (17.1) | 125 (16.8) | 0.883 | 177 (35.3) | 114 (27.1) | 0.008 | 45 (32.6) | 13 (20.0) | 0.064 |
| Age, years | 55.0 [50.0, 60.0] | 57.0 [52.0, 61.0] | <0.001 | 69.0 [67.0, 71.0] | 69.0 [67.0, 72.0] | 0.748 | 77.0 [76.0, 79.0] | 76.0 [75.0, 78.0] | 0.039 |
| **Clinical characteristics** |  |  |  |  |  |  |  |  |  |
| BMI, kg/m^2^ | 26.0 [24.5, 27.7] | 25.9 [24.2, 27.8] | 0.153 | 25.4 [23.4, 27.1] | 25.6 [23.9, 27.1] | 0.221 | 25.0 [22.6, 26.5] | 25.2 [24.0, 26.3] | 0.349 |
| BMI group |  |  |  |  |  |  |  |  |  |
| <18.5 | 3 (0.3) | 4 (0.5) | 0.706 | 8 (1.6) | 4 (1.0) | 0.390 | 2 (1.5) | 3 (4.6) | 0.330 |
| 18.5-24.9 | 281 (29.6) | 268 (36.0) | 0.005 | 210 (41.8) | 165 (39.2) | 0.416 | 67 (48.5) | 27 (41.5) | 0.350 |
| 25.0-29.9 | 583 (61.4) | 399 (53.6) | 0.001 | 249 (49.6) | 224 (53.2) | 0.275 | 64 (46.4) | 30 (46.2) | 0.976 |
| ≥30.0 | 82 (8.6) | 73 (9.8) | 0.407 | 35 (7.0) | 28 (9.7) | 0.847 | 5 (3.6) | 5 (7.7) | 0.211 |
| Smoking history | 596(62.8) | 447 (60.1) | 0.253 | 210 (41.8) | 186 (44.2) | 0.473 | 55 (39.9) | 27 (41.5) | 0.820 |
| Drinking history | 337 (35.5) | 211 (28.4) | 0.002 | 91 (18.1) | 103 (24.5) | 0.019 | 20 (14.5) | 9 (13.8) | 0.902 |
| Comorbidities |  |  |  |  |  |  |  |  |  |
| Diabetes | 298 (31.4) | 256 (34.4) | 0.191 | 198 (39.4) | 141 (33.5) | 0.062 | 39 (28.3) | 18 (27.7) | 0.933 |
| Hypertension | 655(69.0) | 512 (68.8) | 0.929 | 372 (74.1) | 311 (73.9) | 0.936 | 106 (76.8) | 50 (76.9) | 0.986 |
| Dyslipidemia | 583 (61.4) | 435(58.5) | 0.216 | 296 (59.0) | 231 (54.9) | 0.211 | 68 (49.3) | 28 (43.1) | 0.409 |
| Peripheral artery disease | 29 (3.1) | 92 (12.4) | <0.001 | 26 (5.2) | 50 (11.9) | <0.001 | 6 (4.3) | 10 (15.4) | 0.006 |
| COPD | 2 (0.2) | 4 (0.5) | 0.414 | 11 (2.2) | 7 (1.7) | 0.563 | 4 (2.9) | 3 (4.6) | 0.682 |
| Anemia | 3 (0.3) | 0 (0.0) | 0.261 | 3 (0.6) | 3 (0.7) | 1.000 | 2 (1.4) | 0 (0) | 1.000 |
| History |  |  |  |  |  |  |  |  |  |
| Prior MI | 316 (32.3) | 287(38.6) | 0.024 | 150 (29.9) | 150 (35.7) | 0.063 | 39 (28.3) | 24 (36.9) | 0.213 |
| Prior stroke | 74 (7.8) | 73 (9.8) | 0.144 | 59 (11.8) | 50 (11.9) | 0.954 | 15 (10.9) | 7 (10.8) | 0.983 |
| Prior PCI | 122 (12.9) | 82 (11.0) | 0.250 | 69 (13.7) | 34 (8.1) | 0.006 | 19 (13.8) | 4 (6.2) | 0.110 |
| Prior CABG | 34 (3.6) | 5 (0.7) | <0.001 | 29 (5.8) | 2 (0.5) | <0.001 | 8 (5.8) | 1 (1.5) | 0.277 |
| NSTEMI | 158 (16.6) | 78 (10.5) | <0.001 | 78 (15.5) | 58 (13.8) | 0.452 | 33 (23.9) | 7 (10.8) | 0.028 |
| eGFR<60.0 ml/min | 36 (3.8) | 50 (6.7) | 0.006 | 54 (10.8) | 67 (15.9) | 0.021 | 25 (18.1) | 9 (13.8) | 0.447 |
| LVEF<40% | 15 (1.6) | 32 (4.3) | <0.001 | 6 (1.2) | 8 (1.9) | 0.383 | 2 (1.4) | 2 (3.1) | 0.594 |
| Left main involved | 103 (10.9) | 260 (34.9) | <0.001 | 64 (12.7) | 169 (40.1) | <0.001 | 31 (22.5) | 30 (46.2) | <0.001 |
| SYNTAX score | 20.0 [15.0, 26.5] | 30.0[24.0, 36.5] | <0.001 | 21.5 [16.0, 27.0] | 30.5 [23.5, 37.0] | <0.001 | 22.0 [15.5, 29.0] | 32.0 [25.0, 37.0] | <0.001 |
| 0-22 | 577 (60.8) | 151 (20.4) | <0.001 | 277 (55.2) | 86 (20.5) | <0.001 | 69 (50.4) | 11 (16.9) | <0.001 |
| 23-32 | 289 (30.5) | 310 (41.8) | <0.001 | 173 (34.5) | 172 (41.0) | 0.043 | 52 (38.0) | 23 (35.4) | 0.724 |
| ≥33 | 83 (8.7) | 280 (37.8) | <0.001 | 52 (10.4) | 162 (38.6) | <0.001 | 16 (11.7) | 31 (47.7) | <0.001 |
| Post-procedural medication |  |  |  |  |  |  |  |  |  |
| Aspirin | 932(98.2) | 710 (95.4) | <0.001 | 490 (97.6) | 384 (91.2) | <0.001 | 136 (98.6) | 59 (90.8) | 0.014 |
| Satins | 852 (89.8) | 124 (16.7) | <0.001 | 437 (87.1) | 67 (15.9) | <0.001 | 123 (89.1) | 11 (16.9) | <0.001 |
| Beta blockers | 849(89.5) | 660 (88.7) | 0.621 | 444 (88.4) | 356 (84.6) | 0.084 | 118 (85.5) | 48 (73.8) | 0.045 |
| ACEIs/ARBs | 627(66.1) | 80 (10.8) | <0.001 | 333 (66.3) | 69 (16.4) | <0.001 | 96 (69.6) | 12 (18.5) | <0.001 |

Values are expressed as number (%) or median [interquartile range].

PCI, percutaneous coronary intervention; CABG, coronary artery bypass grafting; Std. diff., standardized difference; BMI, body mass index; COPD, chronic obstructive pulmonary disease; MI, myocardial infarction; eGFR, estimated glomerular filtration rate; LVEF, Left ventricular ejection fraction; SYNTAX, synergy between percutaneous coronary intervention with taxus and cardiac surgery; ACEI, angiotensin converting enzyme inhibitor; ARB, angiotensin-receptor blocker.

†Candidate variables selected to generate propensity score.

**Supplementary Table 3 Baseline characteristics according to sex.**

|  | **Women (n=636)** | | | **Men (n=2,183)** | | |
| --- | --- | --- | --- | --- | --- | --- |
|  | **PCI (n=384)** | **CABG (n=252)** | **p** | **PCI (n=1,205)** | **CABG (n=978)** | **p** |
| **Demographic characteristics** | | |  |  |  |  |
| Age^†^, years | 66.0 [60.0, 71.0] | 65.5 [59.8, 70.0] | 0.079 | 60.0 [52.0, 68.0) | 61.0 [55.0, 68.0] | 0.002 |
| Age group |  |  |  |  |  |  |
| <65 years | 162 (42.2) | 125 (49.6) | 0.066 | 787 (65.3) | 619 (63.3) | 0.327 |
| 65-74 years | 177 (46.1) | 114 (45.2) | 0.832 | 325 (27.0) | 307 (31.4) | 0.024 |
| ≥75 years | 45 (11.7) | 13 (5.2) | 0.005 | 91 (7.7) | 52 (5.3) | 0.025 |
| **Clinical characteristics** |  |  |  |  |  |  |
| BMI, kg/m^2^ | 25.2 [23.6, 27.3] | 25.2 [23.6, 27.3] | 0.908 | 26.0 [24.2, 27.6] | 25.9 [24.1, 27.7] | 0.539 |
| BMI group^†^ |  |  |  |  |  |  |
| <18.5 | 7 (1.8) | 1 (0.4) | 0.155 | 6 (0.5) | 10 (1.0) | 0.153 |
| 18.5-24.9 | 153 (39.8) | 101 (40.1) | 0.953 | 405 (33.6) | 359 (36.7) | 0.131 |
| 25.0-29.9 | 188 (49.0) | 132 (52.4) | 0.393 | 708 (58.8) | 521 (53.3) | 0.010 |
| ≥30.0 | 36 (9.4) | 18 (7.1) | 0.323 | 86 (7.1) | 88 (9.0) | 0.110 |
| Smoking history^†^ | 52 (13.5) | 33 (13.1) | 0.871 | 809 (67.1) | 627 (64.1) | 0.138 |
| Drinking history^†^ | 2 (0.5) | 1 (0.4) | 1.000 | 446 (37.0) | 322 (32.9) | 0.047 |
| Comorbidities |  |  |  |  |  |  |
| Diabetes | 170 (44.3) | 98 (38.9) | 0.179 | 365 (30.3) | 317 (32.4) | 0.287 |
| Hypertension^*†^ | 307 (79.9) | 201 (79.8) | 0.954 | 826 (68.5) | 672 (68.7) | 0.935 |
| Dyslipidemia^*†^ | 235 (61.2) | 140 (55.6) | 0.157 | 712 (59.1) | 554 (56.7) | 0.251 |
| Peripheral artery disease^*†^ | 14 (3.6) | 30 (11.9) | <0.001 | 47 (3.9) | 122 (12.5) | <0.001 |
| COPD | 4 (1.0) | 3 (1.2) | 1.000 | 13 (1.1) | 11 (1.1) | 0.919 |
| Anemia | 4 (1.0) | 1 (0.4) | 0.653 | 4 (0.3) | 2 (0.2) | 0.697 |
| History |  |  |  |  |  |  |
| Prior MI^*†^ | 75 (19.5) | 60 (23.8) | 0.197 | 430 (35.7) | 401 (41.0) | 0.011 |
| Prior stroke^*†^ | 37 (9.6) | 33 (13.1) | 0.173 | 111 (9.2) | 97 (9.9) | 0.576 |
| Prior PCI^*†^ | 30 (7.8) | 16 (6.3) | 0.486 | 180 (14.9) | 104 (10.6) | 0.003 |
| Prior CABG^*†^ | 15 (3.9) | 0 (0) | 0.001 | 56 (4.6) | 8 (0.8) | <0.001 |
| NSTEMI | 69 (18.0) | 31 (12.3) | 0.055 | 200 (16.6) | 112 (11.5) | <0.001 |
| eGFR<60.0 ml/min^*†^ | 44 (11.5)) | 30 (11.9) | 0.864 | 71 (5.9) | 96 (9.8) | <0.001 |
| LVEF<40%^*†^ | 6 (1.6) | 9 (3.6) | 0.102 | 17 (1.4) | 33 (3.4) | 0.002 |
| Left main involved^*†^ | 39 (10.2) | 173 (68.7) | <0.001 | 159 (13.2) | 380 (38.9) | <0.001 |
| SYNTAX score^*†^ | 21.0 [16.0, 26.5] | 30.0 [22.5, 35.0] | <0.001 | 21.0 [15.0, 27.0] | 30.5 [24.5, 37.0] | <0.001 |
| 0-22 | 221 (57.6) | 62 (24.6) | <0.001 | 702 (58.3) | 186 (19.1) | <0.001 |
| 23-32 | 128 (33.3) | 103 (40.9) | 0.053 | 386 (32.1) | 402 (41.3) | <0.001 |
| ≥33 | 35 (9.1) | 87 (34.5) | <0.001 | 116 (9.6) | 386 (39.6) | <0.001 |
| Post-procedural medication |  |  |  |  |  |  |
| Aspirin^*†^ | 374 (97.4) | 236 (93.6) | 0.002 | 1,184 (98.3) | 917 (93.8) | <0.001 |
| Satins^*†^ | 335 (87.2) | 44 (17.5) | <0.001 | 1,077 (89.4) | 158 (16.2) | <0.001 |
| Beta blockers^*†^ | 338 (88.0) | 212 (84.1) | 0.160 | 1,073 (89.0) | 852 (87.1) | 0.165 |
| ACEIs/ARBs^*†^ | 267 (69.5) | 38 (15.1) | <0.001 | 789 (65.5) | 123 (12.6) | <0.001 |

†Candidate variables selected to generate propensity score.

Values are expressed as number (%) or median [interquartile range].

PCI, percutaneous coronary intervention; CABG, coronary artery bypass grafting; Std. diff., standardized difference; BMI, body mass index; COPD, chronic obstructive pulmonary disease; MI, myocardial infarction; eGFR, estimated glomerular filtration rate; LVEF, Left ventricular ejection fraction; SYNTAX, synergy between percutaneous coronary intervention with taxus and cardiac surgery; ACEI, angiotensin converting enzyme inhibitor; ARB, angiotensin-receptor blocker.

**Supplementary Table 4 Cumulative incidences of clinical events: PCI versus CABG.**

|  |  | **All patients (n=2,819)** | | | **<65 years (n=1,693)** | | | **65-74 years (n=923)** | | | **≥75 years (n=203)** | | |
| --- | --- | --- | --- | --- | --- | --- | --- | --- | --- | --- | --- | --- | --- |
|  |  | **PCI (n=1,589)** | **CABG**  **(n=1,230)** | **p** | **PCI (n=949)** | **CABG**  **(n=744)** | **p** | **PCI (n=502)** | **CABG**  **(n=421)** | **p** | **PCI (n=138)** | **CABG**  **(n=65)** | **p** |
| MACCE | All | 419 (26.4) | 293 (23.8) | 0.067 | 198 (20.9) | 138 (18.5) | 0.130 | 157 (31.3) | 123 (29.2) | 0.273 | 64 (46.4) | 32 (49.2) | 0.409 |
|  | Women | 106 (27.6) | 65 (25.8) | 0.341 | 29 (17.9) | 22 (17.6) | 0.537 | 60 (33.9) | 35 (30.7) | 0.331 | 17 (37.8) | 8 (61.5) | 0.114 |
|  | Men | 313 (26.0) | 228 (23.3) | 0.163 | 169 (21.5) | 116 (18.7) | 0.115 | 97 (29.8) | 88 (28.7) | 0.406 | 47 (50.5) | 24 (46.2) | 0.370 |
| All-cause death | All | 209 (13.2) | 157 (12.8) | 0.451 | 69 (7.3) | 57 (7.7) | 0.217 | 92 (18.3) | 77 (18.3) | 0.529 | 48 (34.8) | 23 (35.4) | 0.527 |
|  | Women | 56 (14.6) | 35 (13.9) | 0.464 | 14 (8.6) | 6 (4.8) | 0.151 | 31 (17.5) | 23 (20.2) | 0.337 | 11 (24.4) | 6 (46.2) | 0.123 |
|  | Men | 153 (12.7) | 122 (12.5) | 0.403 | 55 (7.0) | 51 (8.2) | 0.217 | 61 (18.8) | 54 (17.6) | 0.390 | 37 (39.8) | 17 (32.7) | 0.253 |
| Myocardial infarction | All | 133 (8.4) | 33 (2.7) | <0.001 | 87 (9.2) | 23 (3.1) | <0.001 | 37 (7.4) | 8 (1.9) | 0.002 | 9 (6.5) | 2 (3.1) | 0.508 |
|  | Women | 26 (6.8) | 8 (3.2) | 0.034 | 8 (4.9) | 5 (4.0) | 0.468 | 15 (8.5) | 2 (1.8) | 0.020 | 3 (6.7) | 1 (7.7) | 1.000 |
|  | Men | 107 (8.9) | 25 (2.6) | <0.001 | 79 (10.0) | 18 (2.9) | <0.001 | 22 (6.8) | 6 (2.0) | <0.001 | 6 (6.5) | 1 (1.9) | 0.422 |
| Stroke | All | 113 (7.1) | 129 (10.5) | 0.001 | 62 (6.5) | 66 (8.9) | 0.044 | 39 (7.8) | 95 (22.6) | 0.010 | 12 (8.7) | 10 (15.4) | 0.119 |
|  | Women | 33 (8.6) | 27 (10.7) | 0.224 | 9 (5.6) | 12 (9.6) | 0.141 | 19 (10.7) | 56 (12.6) | 0.410 | 5 (11.1) | 1 (7.7) | 1.000 |
|  | Men | 80 (6.6) | 102 (10.4) | 0.001 | 53 (6.7) | 54 (8.7) | 0.098 | 20 (6.2) | 39 (12.7) | 0.003 | 7 (7.5) | 9 (17.3) | 0.066 |
| Cardiac death | All | 90 (5.7) | 57 (4.6) | 0.128 | 33 (3.5) | 25 (3.4) | 0.503 | 40 (8.0) | 26 (6.2) | 0.178 | 17 (12.3) | 6 (9.2) | 0.389 |
|  | Women | 32 (8.3) | 12 (4.8) | 0.055 | 8 (4.9) | 1 (0.8) | 0.044 | 17 (9.6) | 9 (7.9) | 0.391 | 7 (15.6) | 2 (15.4) | 1.000 |
|  | Men | 58 (4.8) | 45 (4.6) | 0.449 | 25 (3.2) | 24 (3.9) | 0.285 | 23 (7.1) | 17 (5.5) | 0.265 | 10 (10.8) | 4 (7.7) | 0.348 |
| Unplanned revascularization | All | 211 (13.3) | 37 (3.0) | <0.001 | 136 (14.3) | 26 (3.5) | <0.001 | 61 (12.2) | 11 (2.6) | <0.001 | 14 (10.1) | 0 (0.0) | 0.006 |
|  | Women | 43 (11.2) | 6 (2.4) | <0.001 | 19 (11.7) | 5 (4.0) | 0.014 | 19 (10.7) | 1 (0.9) | 0.001 | 5 (11.1) | 0 (0.0) | 0.577 |
|  | Men | 168 (13.9) | 31 (3.2) | <0.001 | 117 (14.9) | 21 (3.4) | <0.001 | 42 (12.9) | 10 (3.3) | <0.001 | 9 (9.7) | 0 (0.0) | 0.027 |

PCI, percutaneous coronary intervention; CABG, coronary artery bypass grafting; MACCE, major adverse cardiac and cerebrovascular events.

**Supplementary Table 5 Subgroup analysis of MACCE in special population.**

|  | **Number of patients** | **Number of events (%)** | | **IPTW model** | | | **PS adjusted Cox regression model** | | |
| --- | --- | --- | --- | --- | --- | --- | --- | --- | --- |
|  |  | **PCI** | **CABG** | **Adjusted HR [95% CI]** | **p** | **p*** | **Adjusted HR [95% CI]** | **p** | **p*** |
| **NSTEMI** | 412 | 79 (29.4) | 36 (25.2) | 0.651 [0.185-0.990] | 0.047 |  | 0.660 [0.411-1.059] | 0.085 |  |
| Age group |  |  |  |  |  | 0.671 |  |  | 0.213 |
| <65 years | 236 | 34 (21.6) | 10 (12.8) | 0.370 [0.341-0.852] | 0.008 |  | 0.399 [0.161-0.993] | 0.048 |  |
| 65-74 years | 136 | 28 (35.9) | 22(37.9) | 0.940 [0.486-1.817] | 0.853 |  | 0.928 [0.501-1.717] | 0.812 |  |
| ≥75 years | 40 | 17 (51.5) | 4 (57.1) | 1.312 [0.263-6.559] | 0.741 |  | 1.041 [0.234-4.637] | 0.959 |  |
| Sex |  |  |  |  |  | 0.521 |  |  | 0.348 |
| Women | 100 | 22 (31.9) | 9 (29.0) | 0.728 [0.561-3.141] | 0.519 |  | 0.764 [0.436-2.594] | 0.892 |  |
| Men | 312 | 57 (28.5) | 27 (24.1) | 0.980 [0.475-2.019] | 0.956 |  | 0.625 [0.357-1.093] | 0.099 |  |
| **Unstable angina** | 2,407 | 340 (27.6) | 257 (23.6) | 0.635 [0.515-0.784] | <0.001 |  | 0.605 [0.509-0.721] | <0.001 |  |
| Age group |  |  |  |  |  | 0.545 |  |  | 0.888 |
| <65 years | 1,457 | 164 (20.7) | 128 (19.2) | 0.616 [0.468-1.811] | 0.651 |  | 0.638 [0.502-1.810] | 0.472 |  |
| 65-74 years | 787 | 129 (30.4) | 101 (27.8) | 0.670 [0.467-0.960] | 0.029 |  | 0.617 [0.465-0.817] | 0.001 |  |
| ≥75 years | 163 | 47 (44.8) | 28 (48.3) | 1.017 [0.493-1.354] | 0.433 |  | 1.105 [0.413-1.202] | 0.199 |  |
| Sex |  |  |  |  |  | 0.912 |  |  | 0.672 |
| Women | 536 | 84 (26.7) | 56 (25.3) | 0.646 [0.447-1.933] | 0.820 |  | 0.668 [0.468-1.953] | 0.626 |  |
| Men | 1871 | 256 (25.5) | 201 (23.2) | 0.632 [0.494-0.809] | <0.001 |  | 0.611 [0.502-0.743] | <0.001 |  |
| **Diabetes** | 950 | 140 (26.2) | 105 (25.3) | 0.615 [0.455-1.832] | 0.201 |  | 0.778 [0.537-1.128] | 0.186 |  |
| Age group |  |  |  |  |  | 0.280 |  |  | 0.724 |
| <65 years | 554 | 55 (18.5) | 49 (19.1) | 1.551 [0.340-1.893] | 0.061 |  | 0.586 [0.331-1.039] | 0.067 |  |
| 65-74 years | 339 | 66/ (33.3) | 46 (32.6) | 0.655 [0.439-1.077] | 0.338 |  | 0.980 [0.574-1.672] | 0.940 |  |
| ≥75 years | 57 | 19 (48.7) | 10 (55.6) | 1.125 [0.484-2.615] | 0.784 |  | 1.078 [0.313-3.711] | 0.905 |  |
| Sex |  |  |  |  |  | 0.183 |  |  | 0.542 |
| Women | 268 | 52 (30.6) | 29 (29.6) | 0.795 [0.488-1.294] | 0.356 |  | 0.800 [0.510-1.255] | 0.330 |  |
| Men | 682 | 88 (24.1) | 76 (24.0) | 0.560 [0.386-1.312] | 0.279 |  | 0.758 [0.399-1.440] | 0.398 |  |
| **Non-diabetes** | 1,869 | 279 (26.5) | 188 (23.1) | 0.715 [0.552-0.927] | 0.011 |  | 0.643 [0.482-0.859] | 0.003 |  |
| Age group |  |  |  |  |  | 0.851 |  |  | 0.239 |
| <65 years | 1,139 | 143 (22.0) | 89 (18.2) | 0.718 [0.506-1.018] | 0.063 |  | 0.656 [0.435-0.987] | 0.043 |  |
| 65-74 years | 584 | 91 (29.9) | 77 (27.5) | 0.750 [0.486-1.157] | 0.194 |  | 0.658 [0.412-1.051] | 0.080 |  |
| ≥75 years | 146 | 45 (45.5) | 22 (46.8) | 0.846 [0.472-1.517] | 0.574 |  | 0.752 [0.317-1.782] | 0.517 |  |
| Sex |  |  |  |  |  | 0.799 |  |  | 0.484 |
| Women | 368 | 54 (25.2) | 36 (23.4) | 0.688 [0.427-1.110] | 0.126 |  | 0.617 [0.337-1.128] | 0.116 |  |
| Men | 1,501 | 225 (26.8) | 152 (23.0) | 0.723 [0.539-0.971] | 0.031 |  | 0.655 [0.471-0.910] | 0.012 |  |
| **Left main involved** | 657 | 53 (26.8) | 120 (26.1) | 0.740 [0.508-1.078] | 0.117 |  | 0.735 [0.535-1.010] | 0.058 |  |
| Age group |  |  |  |  |  | 0.789 |  |  | 0.635 |
| <65 years | 363 | 25 (24.3) | 54 (20.8) | 0.770 [0.421-1.410] | 0.397 |  | 0.765 [0.480-1.220] | 0.261 |  |
| 65-74 years | 233 | 16 (25.0) | 52 (30.8) | 0.808 [0.469-1.394] | 0.444 |  | 0.792 [0.469-1.337] | 0.382 |  |
| ≥75 years | 61 | 12 (38.7) | 14 (46.7) | 1.097 [0.692-1.221] | 0.158 |  | 1.075 [0.199-1.134] | 0.094 |  |
| Sex |  |  |  |  |  | 0.105 |  |  | 0.259 |
| Women | 116 | 7 (17.9) | 22 (27.8) | 1.312 [0.543-3.171] | 0.547 |  | 1.060 [0.489-2.296] | 0.883 |  |
| Men | 539 | 46 (28.9) | 98 (25.8) | 0.680 [0.452-1.022] | 0.063 |  | 0.671 [0.473-0.951] | 0.025 |  |
| **Left main uninvolved** | 2,162 | 366 (26.3) | 173 (22.4) | 0.661 [0.521-0.840] | 0.001 |  | 0.597 [0.494-0.722] | <0.001 |  |
| Age group |  |  |  |  |  | 0.133 |  |  | 0.155 |
| <65 years | 1,330 | 173 (20.4) | 84 (17.4) | 0.630 [0.453-0.875] | 0.006 |  | 0.574 [0.438-0.754] | <0.001 |  |
| 65-74 years | 690 | 141 (32.2) | 71 (28.2) | 0.680 [0.463-1.001] | 0.051 |  | 0.623 [0.462-0.840] | 0.002 |  |
| ≥75 years | 142 | 52 (48.6) | 18 (51.4) | 1.107 [0.601-2.038] | 0.745 |  | 0.876 [0.488-1.572] | 0.657 |  |
| Sex |  |  |  |  |  | 0.777 |  |  | 0.344 |
| Women | 518 | 99 (28.7) | 43 (24.9) | 0.632 [0.427-0.936] | 0.022 |  | 0.631 [0.436-0.915] | 0.015 |  |
| Men | 1,644 | 267 (25.5) | 130 (21.7) | 0.670 [0.503-0.893] | 0.006 |  | 0.591 [0.474-0.736] | <0.001 |  |
| **SYNTAX score 0-22** | 1,171 | 233 (25.2) | 55 (22.2) | 0.782 [0.546-1.120] | 0.180 |  | 0.691 [0.521-0.916] | 0.010 |  |
| Age group |  |  |  |  |  | **0.015** |  |  | **0.027** |
| <65 years | 728 | 119 (20.6) | 30 (19.9) | 0.734 [0.453-1.190] | 0.210 |  | 0.706 [0.480-1.037] | 0.076 |  |
| 65-74 years | 363 | 87 (31.4) | 18 (20.9) | 0.711 [0.394-1.282] | 0.257 |  | 0.543 [0.337-0.875] | 0.012 |  |
| ≥75 years | 80 | 27 (39.1) | 7 (63.6) | 2.014 [0.846-4.810] | 0.115 |  | 1.887 [0.797-4.468] | 0.149 |  |
| Sex |  |  |  |  |  | 0.264 |  |  | 0.719 |
| Women | 283 | 56 (25.3) | 12 (19.4) | 0.618 [0.334-1.145] | 0.126 |  | 0.541 [0.290-1.006] | 0.052 |  |
| Men | 888 | 177 (25.2) | 43 (23.1) | 0.835 [0.550-1.268] | 0.397 |  | 0.732 [0.533-1.006] | 0.055 |  |
| **SYNTAX score 23-32** | 1,019 | 139 (27.0) | 102 (20.2) | 0.579 [0.448-0.749] | <0.001 |  | 0.558 [0.433-0.718] | <0.001 |  |
| Age group |  |  |  |  |  | 0.564 |  |  | 0.422 |
| <65 years | 599 | 61 (21.1) | 44 (14.2) | 0.539 [0.367-0.791] | 0.002 |  | 0.506 [0.348-0.734] | <0.001 |  |
| 65-74 years | 345 | 52 (30.1) | 49 (28.5) | 0.724 [0.492-1.066] | 0.102 |  | 0.708 [0.476-1.053] | 0.089 |  |
| ≥75 years | 75 | 26 (50.0) | 9 (39.1) | 0.603 [0.258-1.406] | 0.242 |  | 0.701 [0.320-1.536] | 0.375 |  |
| Sex |  |  |  |  |  | 0.505 |  |  | 0.537 |
| Women | 231 | 40 (31.3) | 24 (23.3) | 0.670 [0.400-1.124] | 0.129 |  | 0.685 [0.411-1.144] | 0.148 |  |
| Men | 788 | 99 (25.6) | 78 (19.4) | 0.553 [0.412-0.743] | <0.001 |  | 0.519 [0.387-0.694] | <0.001 |  |
| **SYNTAX score** **≥33** | 624 | 47 (31.1) | 136 (28.8) | 0.655 [0.453-0.949] | 0.025 |  | 0.664 [0.486-0.907] | 0.010 |  |
| Age group |  |  |  |  |  | 0.383 |  |  | 0.692 |
| <65 years | 363 | 18 (21.7) | 64 (22.9) | 0.716 [0.398-1.288] | 0.264 |  | 0.715 [0.448-1.142] | 0.160 |  |
| 65-74 years | 214 | 18 (34.6) | 56 (34.6) | 0.667 [0.387-1.149] | 0.145 |  | 0.723 [0.433-1.208] | 0.216 |  |
| ≥75 years | 47 | 11 (68.8) | 16 (51.6) | 0.340 [0.162-0.716] | 0.005 |  | 0.310 [0.138-0.701] | 0.005 |  |
| Sex |  |  |  |  |  | 0.170 |  |  | 0.373 |
| Women | 122 | 10 (28.6) | 29 (33.3) | 0.968 [0.467-2.005] | 0.929 |  | 0.872 [0.447-1.699] | 0.687 |  |
| Men | 502 | 37 (31.9) | 107 (27.7) | 0.599 [0.395-0.908] | 0.016 |  | 0.611 [0.429-0.870] | 0.006 |  |

^*^Interaction p.

MACCE, major adverse cardiac and cerebrovascular events; PCI, percutaneous coronary intervention; CABG, coronary artery bypass grafting; IPTW, inverse probability of treatment weighting; PS, propensity score; HR, hazard ratio; CI, confidence interval; NSTEMI, non-ST-segment elevation myocardial infarction; SYNTAX, synergy between percutaneous coronary intervention with taxus and cardiac surgery.

**Supplementary Table 6 E-value for IPTW adjusted hazard ratio and limit of the confidence interval closest to the null.**

|  |  | **All patients** | | **<65 years** | | **65-74 years** | | **≥75years** | |
| --- | --- | --- | --- | --- | --- | --- | --- | --- | --- |
|  |  | **E-value^*^** | **E-value^†^** | **E-value^*^** | **E-value^†^** | **E-value^*^** | **E-value^†^** | **E-value^*^** | **E-value^†^** |
| MACCE | All | 1.95 | 1.54 | 1.99 | 1.40 | 1.88 | 1.21 | - | - |
|  | Women | - | - | - | - | - | - | 3.92 | 1.87 |
|  | Men | 1.97 | 1.49 | 1.96 | 1.28 | - | - | - | - |
| All-cause death | All | - | - | - | - | - | - | - | - |
|  | Women | - | - | 6.93 | 1.91 | - | - | 3.55 | 1.12 |
|  | Men | - | - | - | - | - | - | - | - |
| Myocardial infarction | All | 6.24 | 3.77 | 4.87 | 2.56 | 10.71 | 3.99 | - | - |
|  | Women | - | - | - | - | - | - | - | - |
|  | Men | 6.99 | 3.97 | 5.75 | 2.87 | 12.05 | 4.38 | - | - |
| Stroke | All | 3.15 | 1.92 | 2.84 | 1.34 | 3.70 | 1.67 | - | - |
|  | Women | - | - | - | - | - | - | - | - |
|  | Men | 3.66 | 2.08 | 2.91 | 1.21 | 5.59 | 2.22 | - | - |
| Cardiac death | All | 3.08 | 1.57 | - | - | - | - | - | - |
|  | Women | - | - | 16.86 | 1.42 | - | - | - | - |
|  | Men | 3.09 | 1.27 | - | - | - | - | - | - |
| Unplanned revascularization | All | 8.15 | 5.21 | 7.25 | 4.17 | 10.15 | 4.44 | 21.32 | 2.07 |
|  | Women | 9.15 | 3.06 | 6.09 | 1.16 | 37.37 | 4.40 | - | - |
|  | Men | 7.96 | 4.84 | 7.53 | 4.09 | 8.21 | 3.27 | - | - |

^*^ E-value for hazard ratio.

^†^ E-value for limit of the confidence interval closest to the null.

E-value is not calculated if the confidence interval includes the null.

IPTW, inverse probability of treatment weighting; MACCE, major adverse cardiac and cerebrovascular events.

**Supplementary Table 7 The largest covariate-outcome associations for each outcome.**

|  |  | **All patients** | | **<65 years** | | **65-74 years** | | **≥75years** | |
| --- | --- | --- | --- | --- | --- | --- | --- | --- | --- |
|  |  | **Covariate** | **HR [95% CI]** | **Covariate** | **HR [95% CI]** | **Covariate** | **HR [95% CI]** | **Covariate** | **HR [95% CI]** |
| MACCE | All | Aspirin^*^ | 1.736 [1.294-2.331] | Aspirin^*^ | 1.621 [1.011-2.604] | Aspirin^*^ | 1.776 [1.182-2.674] | - | - |
|  | Women | - | - | - | - | - | - | Aspirin^*^ | 1.669 [1.098-2.545] |
|  | Men | Aspirin^*^ | 1.923 [1.387-2.667] | Aspirin^*^ | 1.789 [1.098-2.545] | - | - | - | - |
| All-cause death | All | - | - | - | - | - | - | - | - |
|  | Women | - | - | LVEF<40% | 4.531 [2.391-8.585] | - | - | LVEF<40% | 4.531 [2.391-8.585] |
|  | Men | - | - | - | - | - | - | - | - |
| Myocardial infarction | All | Statin | 2.885 [1.978-4.207] | Statin | 2.283 [1.467-3.551] | Statin | 4.783 [2.142-10.680] | - | - |
|  | Women | - | - | - | - | - | - | - | - |
|  | Men | Statin | 3.155 [2.053-4.846] | Statin | 3.956 [2.184-7.165] | Statin | 2.232 [1.364-3.655] | - | - |
| Stroke | All | Hypertension | 1.716 [1.248-2.359] | Hypertension | 1.860 [1.201-2.879] | NSTEMI | 1.767 [1.056-2.950] | - | - |
|  | Women | - | - | - | - | - | - | - | - |
|  | Men | Hypertension | 1.580 [1.116-2.238] | Diabetes | 1.500 [1.053-2.137] | Hypertension | 2.355 [1.426-3.891] | - | - |
| Cardiac death | All | LVEF<40% | 4.346 [2.212-8.540] | - | - | - | - | - | - |
|  | Women | - | - | LVEF<40% | 5.750 [2.309-14.314] | - | - | - | - |
|  | Men | LVEF<40% | 6.697 [3.373-13.297] | - | - | - | - | - | - |
| Unplanned  revascularization | All | Statin^*^ | 3.057 [2.234-4.183] | Statin^*^ | 2.692 [1.838-3.943] | Statin^*^ | 3.400 [1.933-5.981] | Prior PCI | 3.383 [1.059-10.810] |
|  | Women | Statin^*^ | 2.950 [1.429-6.090] | Statin^*^ | 2.870 [1.874-4.395] | Statin^*^ | 3.294 [2.071-5.237] | - | - |
|  | Men | Statin^*^ | 3.108 [2.195-4.401] | Statin^*^ | 2.870 [1.874-4.395] | Statin^*^ | 3.294 [2.071-5.237] | - | - |

^*^ Inverted for protective associations.

HR, hazard ratio; CI, confidence interval; MACCE, major adverse cardiac and cerebrovascular events.

**Supplementary Table 8 PS adjusted risks of CABG relative to PCI for clinical events.**

|  |  | **All patients (n=2812)** | | **<65 years (n=1677)** | | **65-74 years (n=932)** | | **≥75 years (n=203)** | | **p^*^** |
| --- | --- | --- | --- | --- | --- | --- | --- | --- | --- | --- |
|  |  | **Adjusted HR [95% CI]** | **p** | **Adjusted HR [95% CI]** | **p** | **Adjusted HR [95% CI]** | **p** | **Adjusted HR [95% CI]** | **p** |  |
| MACCE | All | 0.692 [0.551-0.869] | 0.002 | 0.634 [0.454-0.884] | 0.007 | 0.783 [0.551-1.113] | 0.172 | 0.824 [0.404-1.680] | 0.593 | 0.209 |
|  | Women | 0.677 [0.436-1.050] | 0.082 | 0.402 [0.183-0.885] | 0.024 | 0.744 [0.415-1.333] | 0.320 | 3.463 [0.970-12.357] | 0.056 | 0.062 |
|  | Men | 0.703 [0.538-0.918] | 0.010 | 0.696 [0.483-1.004] | 0.053 | 0.818 [0.526-1.272] | 0.373 | 0.460 [0.194-1.091] | 0.078 | 0.872 |
|  | p^†^ |  | 0.240 |  | 0.878 |  | 0.805 |  | **0.027** |  |
| All-cause death | All | 0.900 [0.637-1.272] | 0.550 | 1.122 [0.612-2.056] | 0.710 | 1.087 [0.670-1.764] | 0.736 | 0.447 [0.189-1.056] | 0.068 | 0.999 |
|  | Women | 0.902 [0.474-1.715] | 0.752 | 0.439 [0.091-2.119] | 0.305 | 1.234 [0.564-2.699] | 0.599 | 1.615 [0.328-7.952] | 0.556 | 0.063 |
|  | Men | 0.913 [0.605-1.377] | 0.665 | 1.361 [0.735-2.639] | 0.362 | 1.049 [0.569-1.935] | 0.878 | 0.267 [0.099-0.724] | 0.009 | 0.417 |
|  | p^†^ |  | 0.362 |  | 0.063 |  | 0.301 |  | **0.047** |  |
| Myocardial infarction | All | 0.466 [0.263-0.824] | 0.009 | 0.460 [0.226-0.938] | 0.033 | 0.414 [0.144-1.190] | 0.102 | 0.983 [0.092-10.492] | 0.989 | 0.514 |
|  | Women | 0.602 [0.195-1.855] | 0.376 | 0.667 [0.114-3.890] | 0.652 | 0.297 [0.045-1.940] | 0.205 | 9.121 [0.235-354.456] | 0.237 | 0.205 |
|  | Men | 0.432 [0.224-0.833] | 0.012 | 0.422 [0.193-0.925] | 0.031 | 0.495 [0.134-1.829] | 0.292 | 0.212 [0.005-8.637] | 0.412 | 0.898 |
|  | p^†^ |  | 0.331 |  | 0.131 |  | 0.782 |  | 0.346 |  |
| Stroke | All | 1.319 [0.864-2.013] | 0.199 | 0.841 [0.467-1.515] | 0.564 | 2.063 [1.090-3.904] | 0.026 | 2.860 [0.720-11.358] | 0.135 | 0.601 |
|  | Women | 0.674 [0.310-1.467] | 0.312 | 0.363 [0.102-1.293] | 0.118 | 0.915 [0.330-2.538] | 0.865 | 3.362 [0.113-100.298] | 0.484 | 0.747 |
|  | Men | 1.719 [1.047-2.823] | 0.032 | 1.025 [0.533-1.972] | 0.941 | 3.526 [1.579-7.876] | 0.002 | 2.763 [0.558-13.676] | 0.213 | 0.348 |
|  | p^†^ |  | 0.336 |  | 0.735 |  | 0.112 |  | 0.411 |  |
| Cardiac death | All | 0.491 [0.295-0.818] | 0.006 | 1.031 [0.426-2.494] | 0.946 | 0.517 [0.254-1.052] | 0.069 | 0.118 [0.033-0.423] | 0.001 | 0.826 |
|  | Women | 0.468 [0.180-1.221] | 0.121 | 0.339 [0.018-6.490] | 0.473 | 0.783 [0.251-2.447] | 0.674 | 0.332 [0.037-3.007] | 0.327 | 0.213 |
|  | Men | 0.492 [0.267-0.908] | 0.023 | 1.220 [0.468-3.185] | 0.684 | 0.398 [0.161-0.980] | 0.045 | 0.062 [0.014-0.282] | <0.001 | 0.438 |
|  | p^†^ |  | 0.414 |  | **0.025** |  | 0.801 |  | 0.555 |  |
| Cardiac  rehospitalization | All | 0.532 [0.379-0.747] | <0.001 | 0.486 [0.249-0.946] | 0.034 | 0.566 [0.333-0.960] | 0.035 | 1.414 [0.453-4.410] | 0.551 | 0.618 |
|  | Women | 0.502 [0.261-0.964] | 0.038 | 0.368 [0.127-1.066] | 0.066 | 0.479 [0.193-1.191] | 0.113 | 2.471 [0.344-17.757] | 0.369 | 0.676 |
|  | Men | 0.545 [0.366-0.810] | 0.003 | 0.469 [0.279-0.791] | 0.005 | 0.604 [0.312-1.170] | 0.135 | 1.058 [0.249-4.490] | 0.940 | 0.557 |
|  | p^†^ |  | 0.967 |  | 0.547 |  | 0.382 |  | 0.900 |  |
| Unplanned revascularization | All | 0.282 [0.169-0.473] | <0.001 | 0.279 [0.145-0.537] | <0.001 | 0.234 [0.096-0.568] | 0.001 | 0.771 [0.071-8.401] | 0.831 | 0.780 |
|  | Women | 0.310 [0.097-0.988] | 0.048 | 0.249 [0.050-1.225] | 0.087 | 0.108 [0.011-1.090] | 0.059 | 0.190 [0.405-68.562] | 0.205 | 0.312 |
|  | Men | 0.274 [0.154-0.488] | <0.001 | 0.285 [0.139-0.583] | 0.001 | 0.264 [0.097-0.720] | 0.009 | - | - | 0.316 |
|  | p^†^ |  | 0.871 |  | 0.845 |  | 0.288 |  | 0.092 |  |

PS, propensity score; CABG, coronary artery bypass grafting; PCI, percutaneous coronary intervention; HR, hazard ratio; CI, confidence interval; MACCE, major adverse cardiac and cerebrovascular events.

**Supplementary Figures**


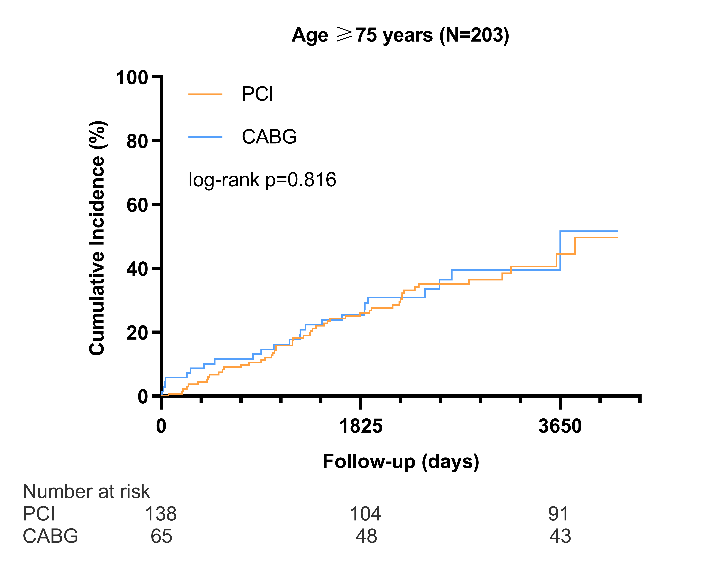

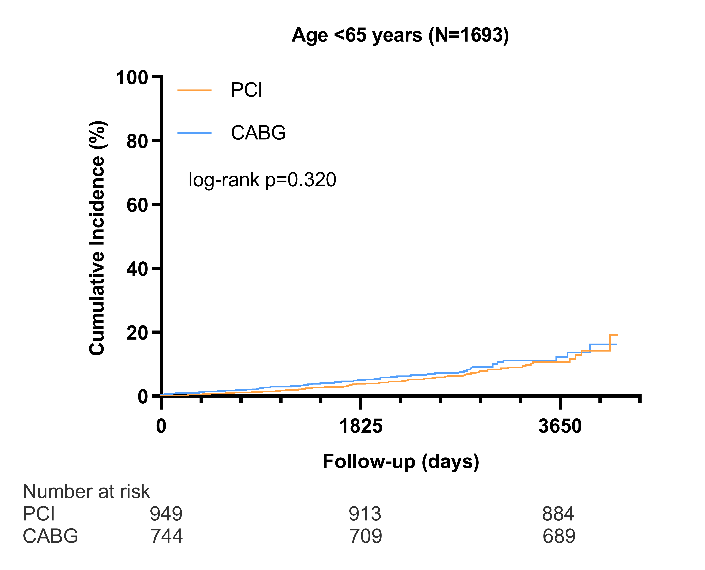

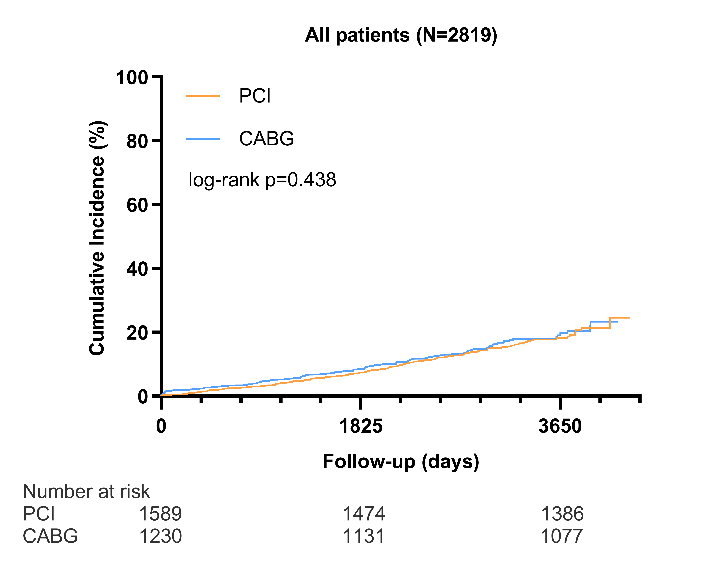

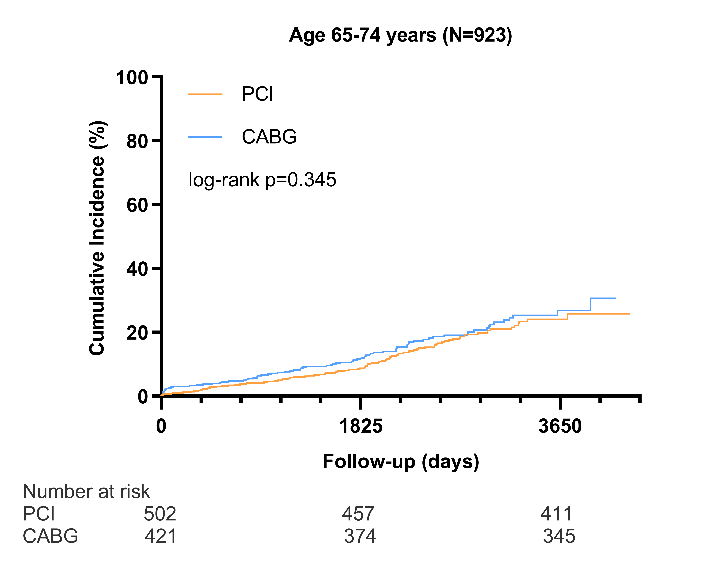

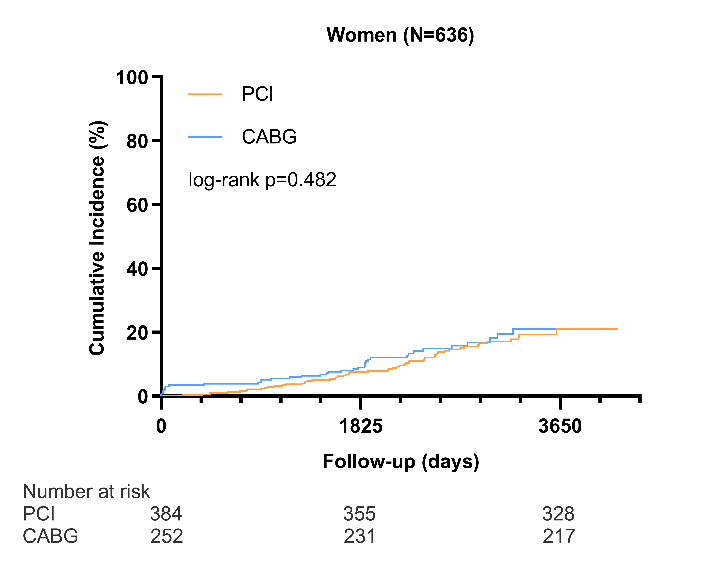

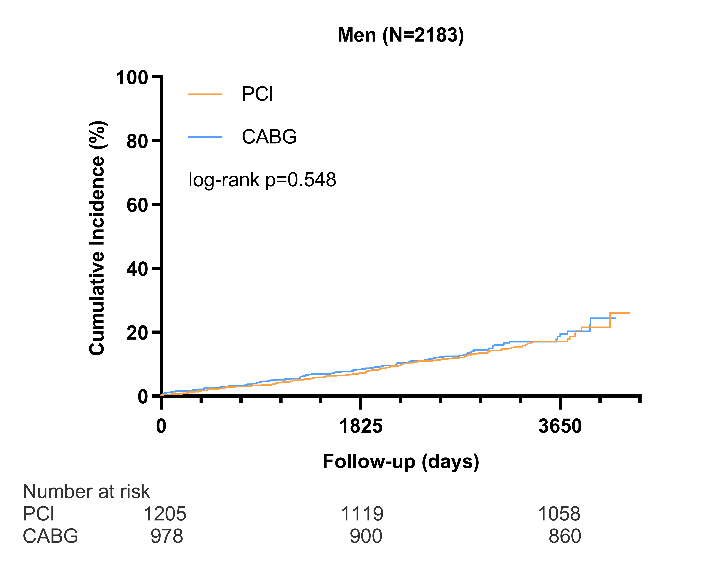


**Supplementary Figure 1 Cumulative incidence of all-cause death.**

PCI, percutaneous coronary intervention; CABG, coronary artery bypass grafting.

**Supplementary Figure 2 Cumulative incidence of myocardial infarction.**


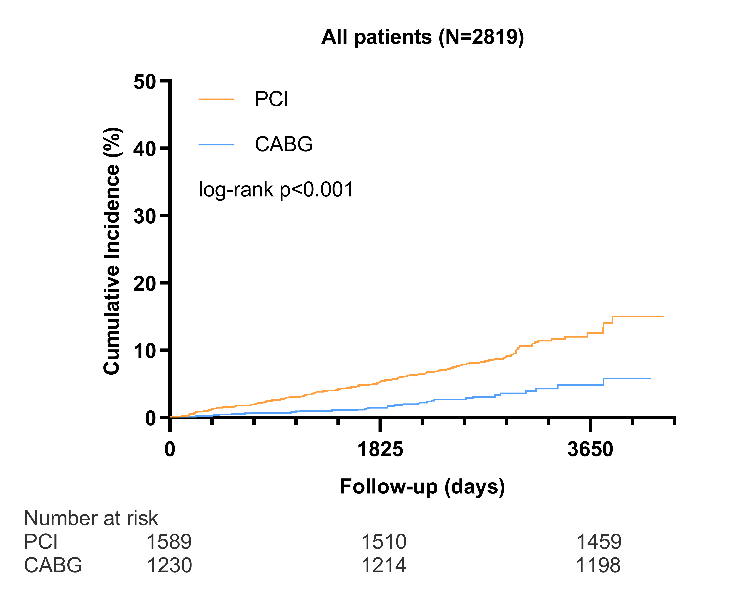

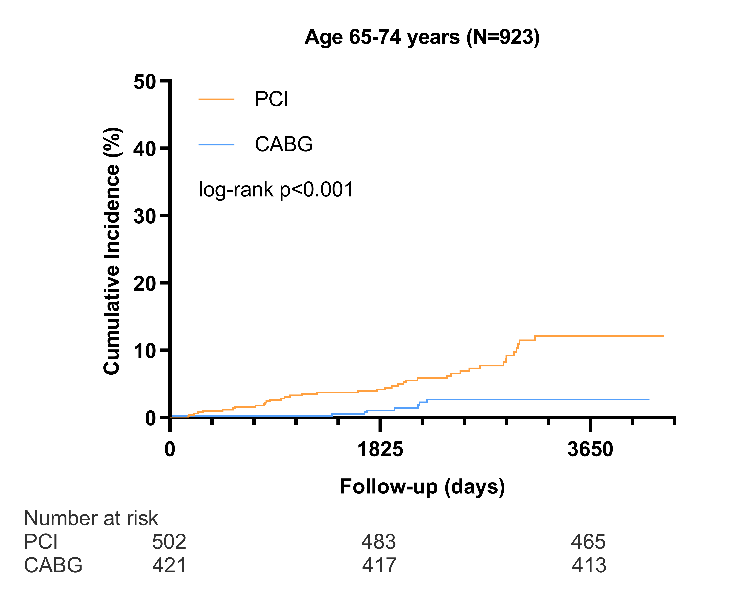

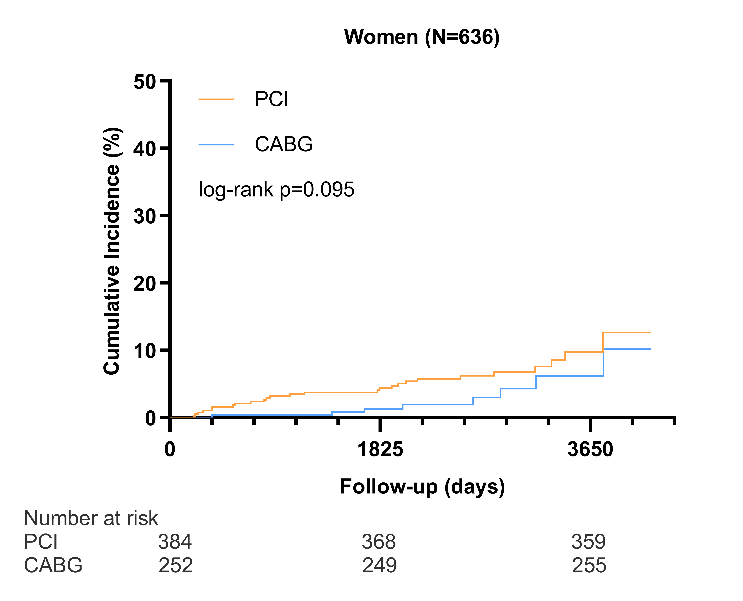

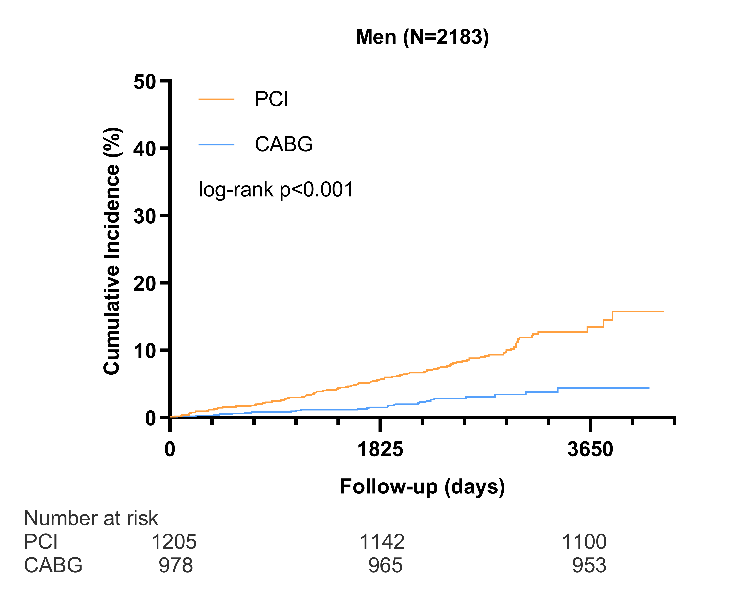

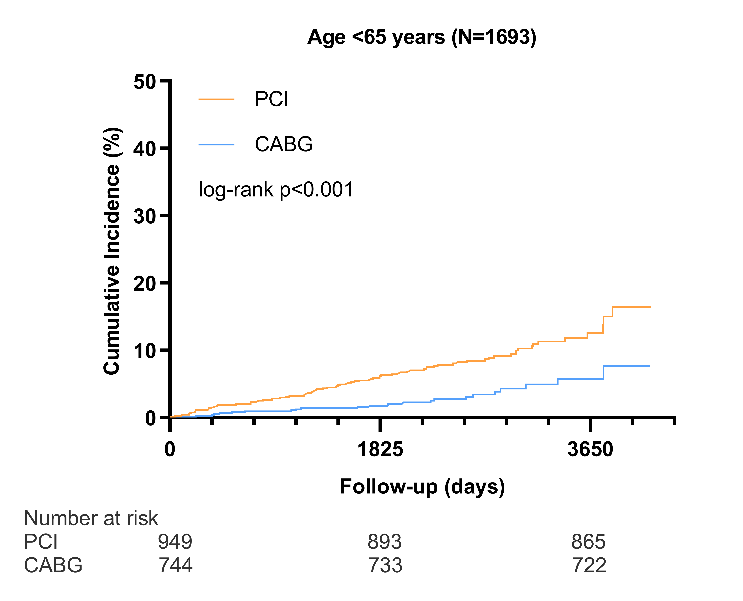

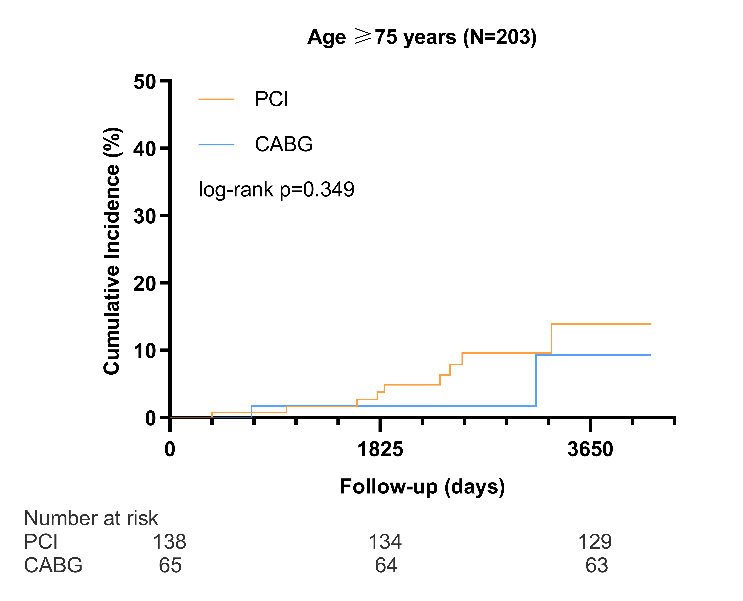


PCI, percutaneous coronary intervention; CABG, coronary artery bypass grafting.

**Supplementary Figure 3 Cumulative incidence of stroke.**


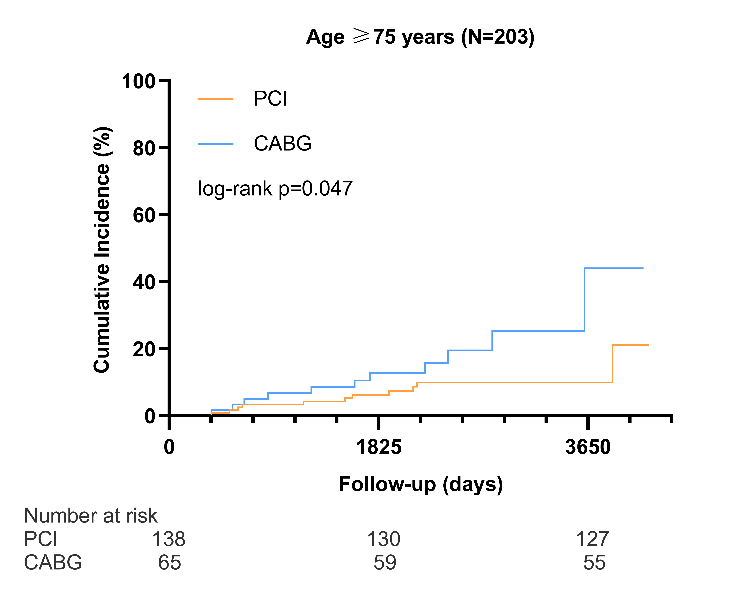

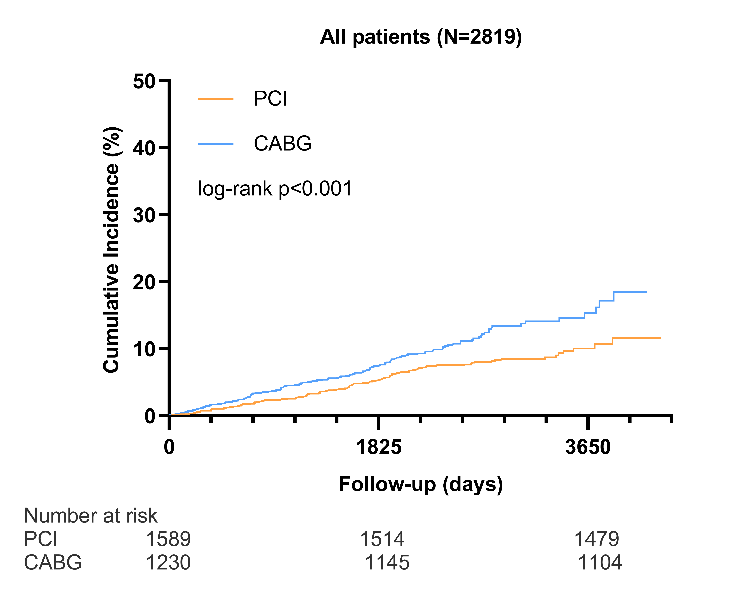

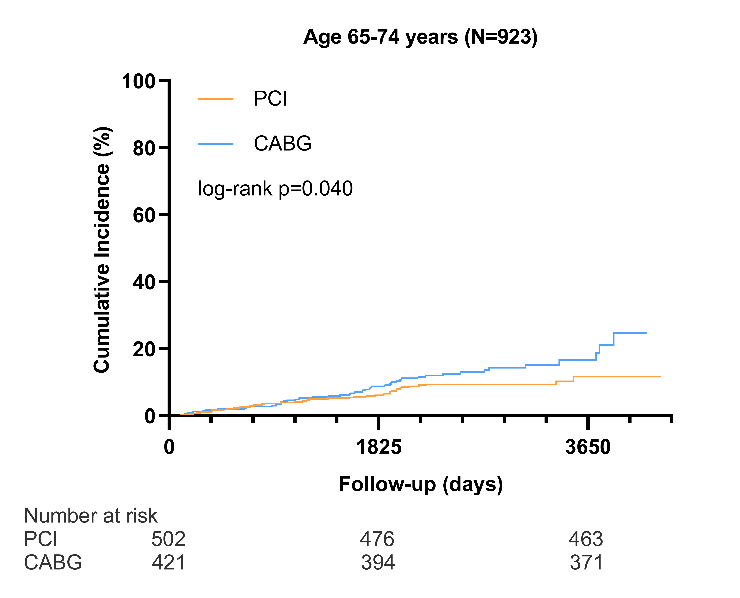

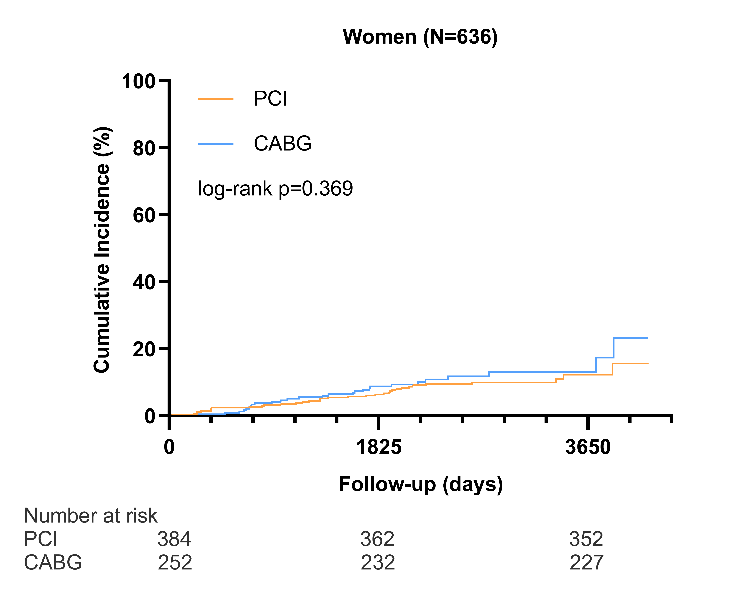

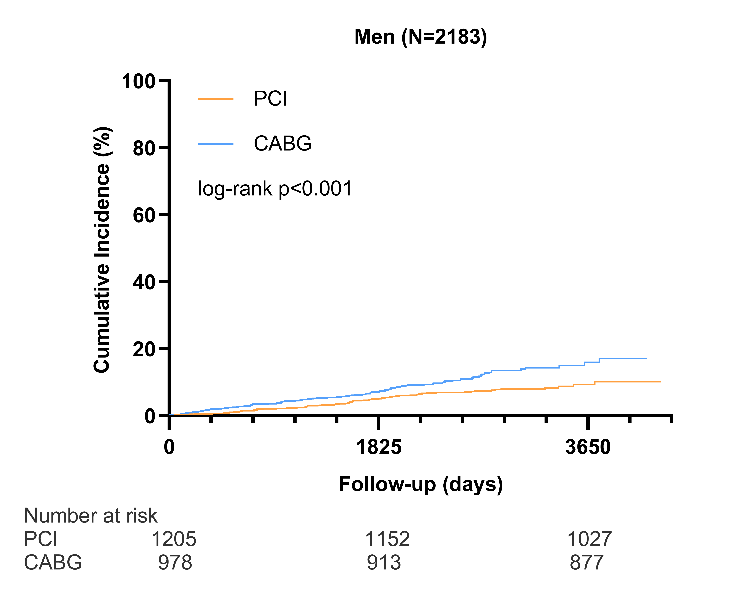

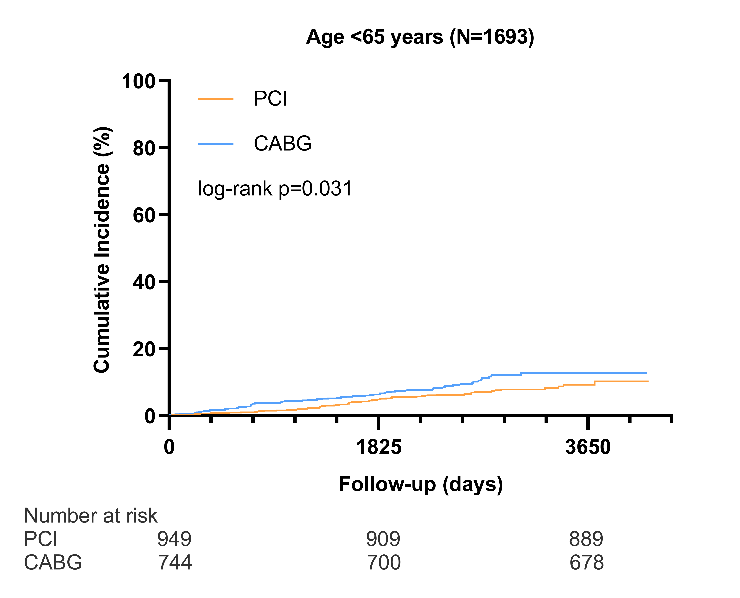


PCI, percutaneous coronary intervention; CABG, coronary artery bypass grafting.

**Supplementary Figure 4 Cumulative incidence of cardiac death.**


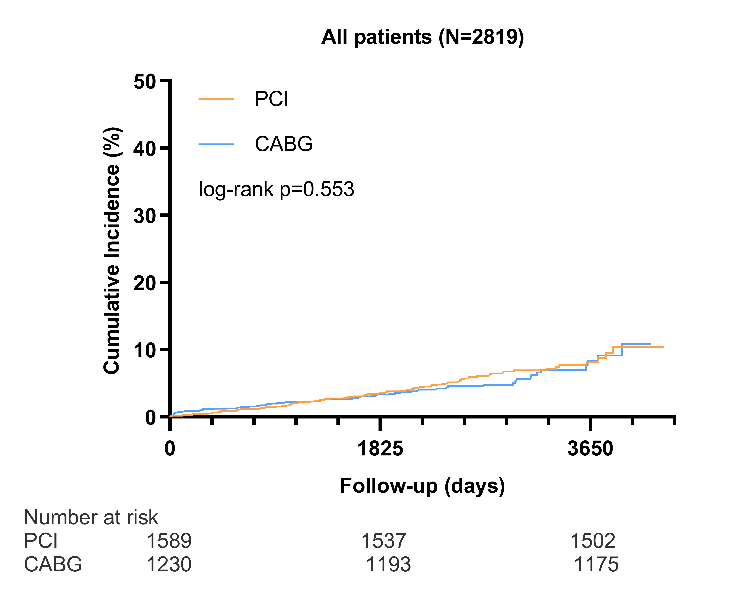

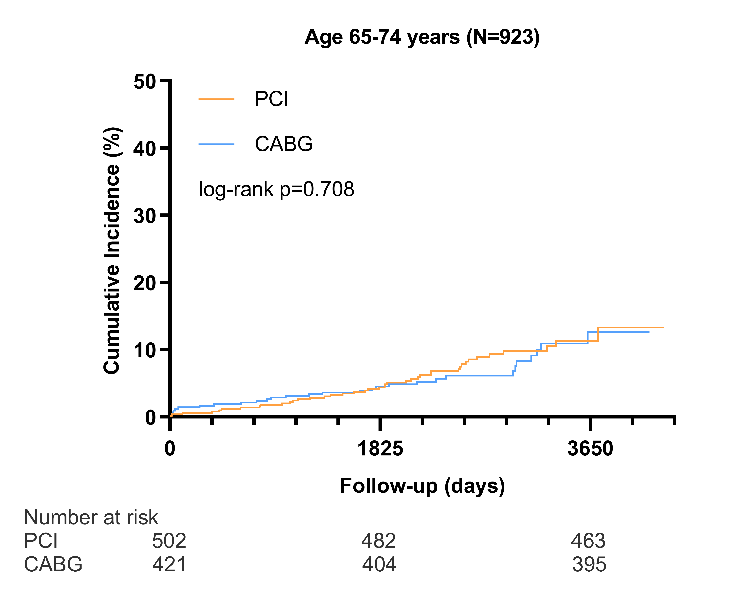

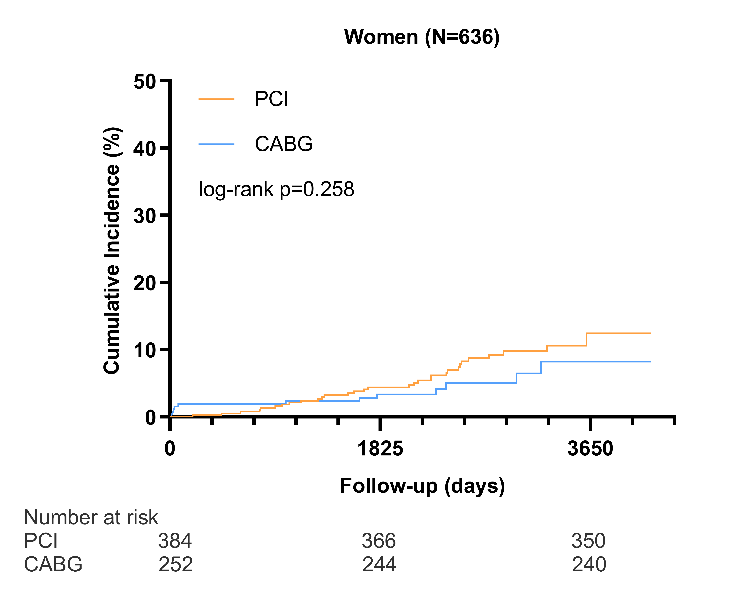

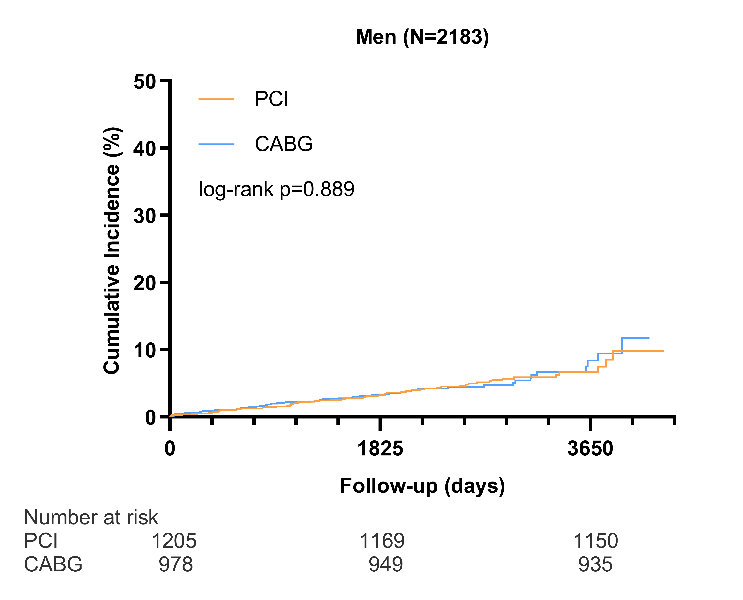

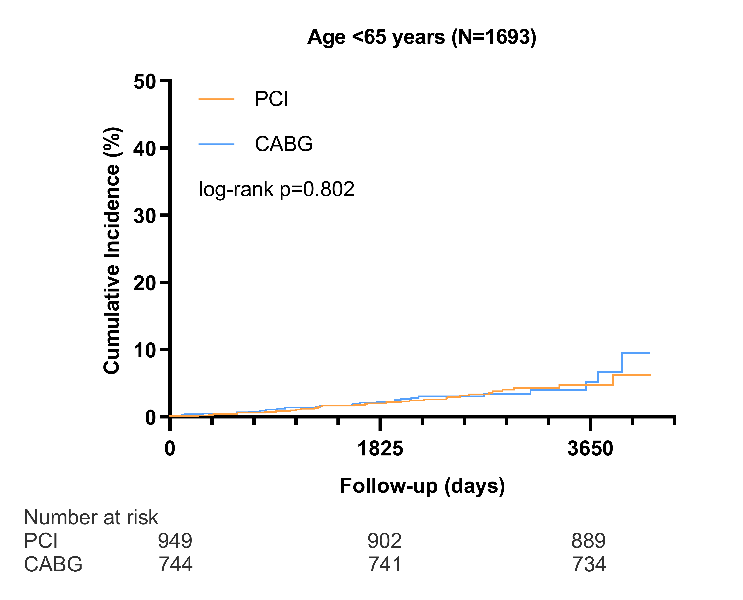

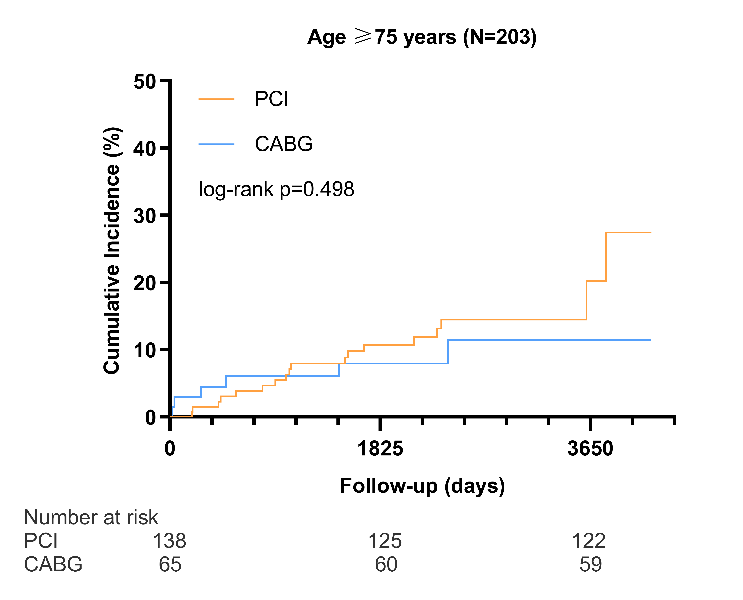


PCI, percutaneous coronary intervention; CABG, coronary artery bypass grafting.

**Supplementary Figure 5 Cumulative incidence of unplanned revascularization.**


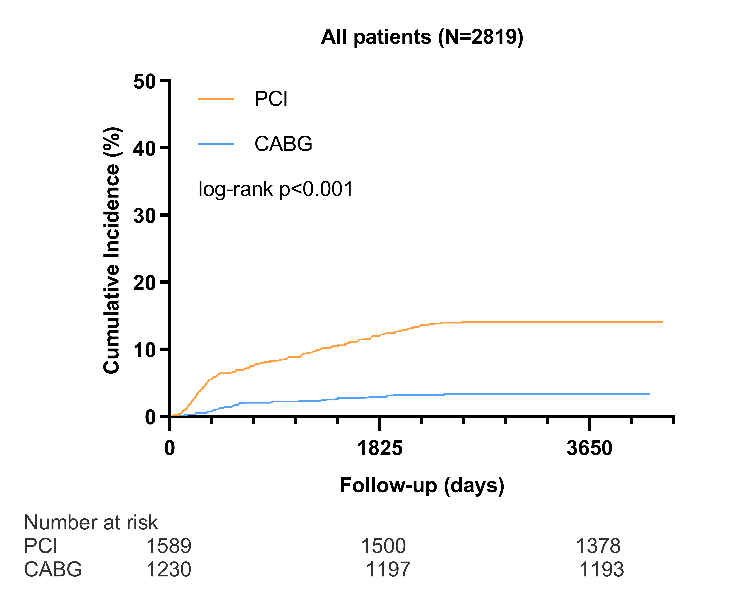

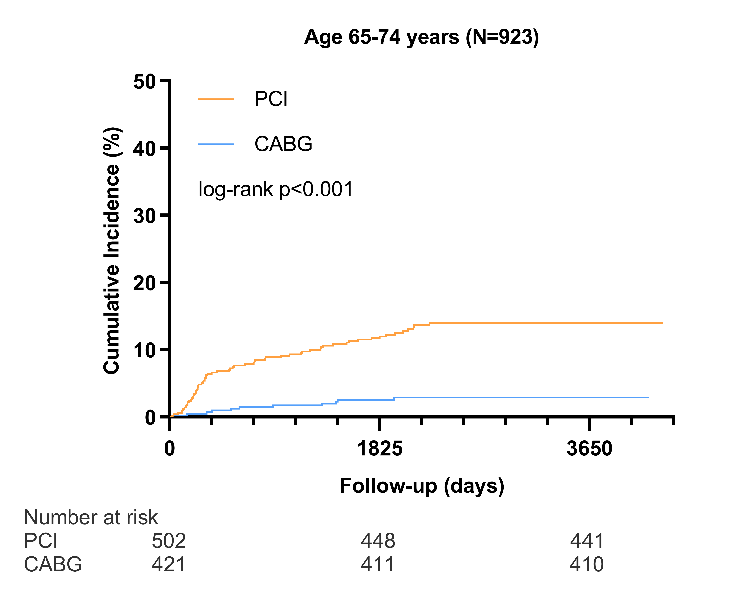

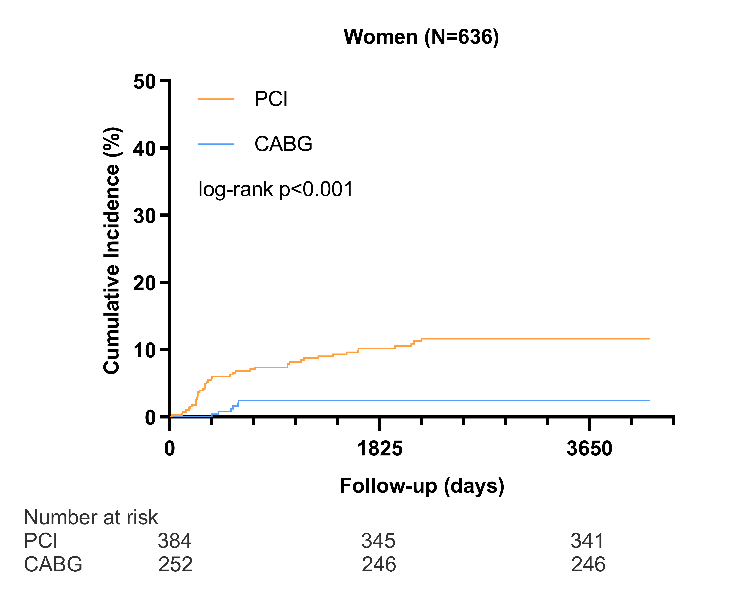

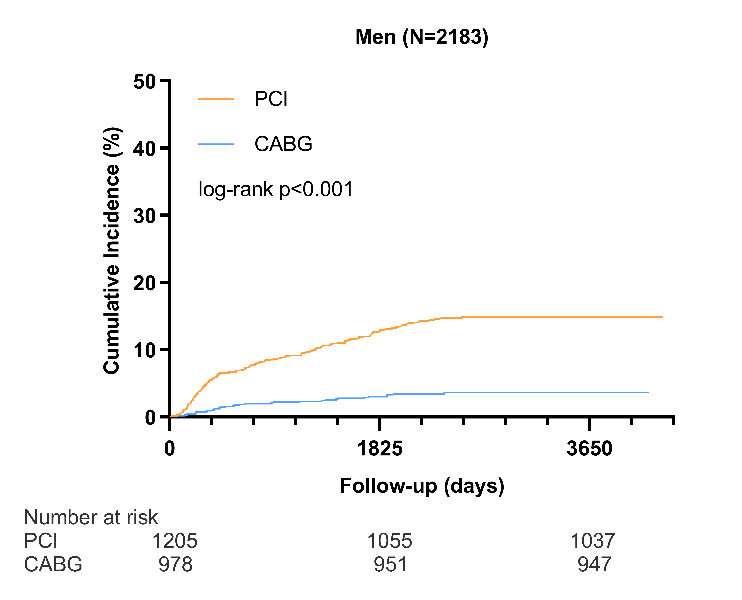

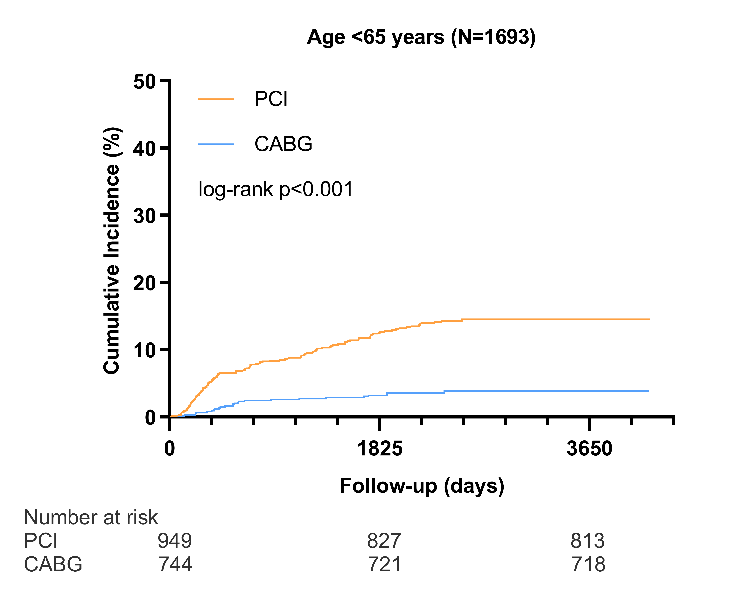

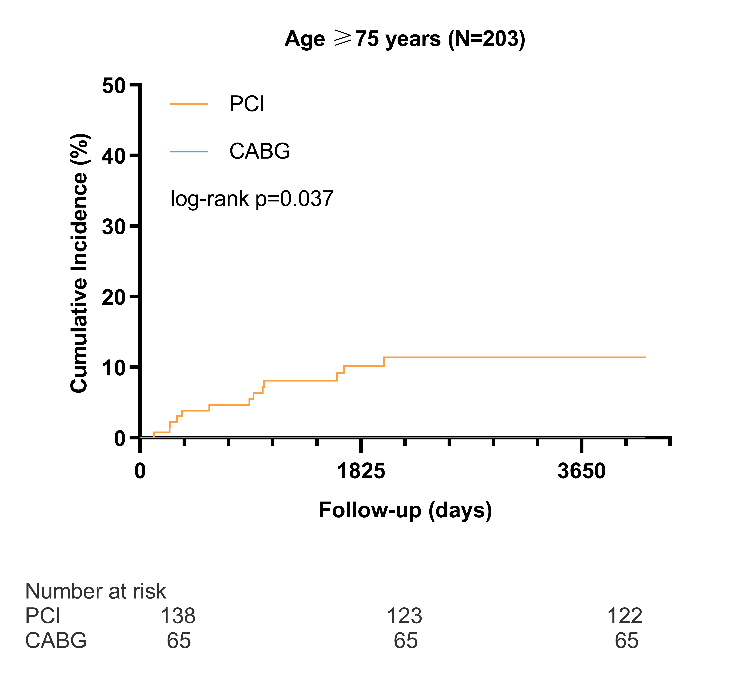


PCI, percutaneous coronary intervention; CABG, coronary artery bypass grafting.

**Supplementary Figure 6 IPTW adjusted hazard ratio of CABG relative to PCI for MACCE stratified by age group and sex in subgroups.**


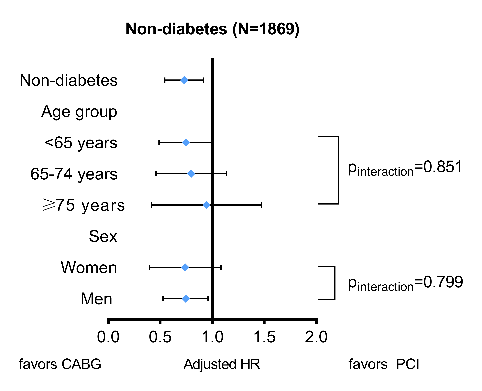

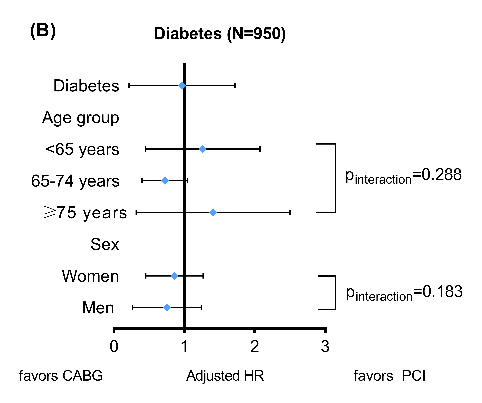

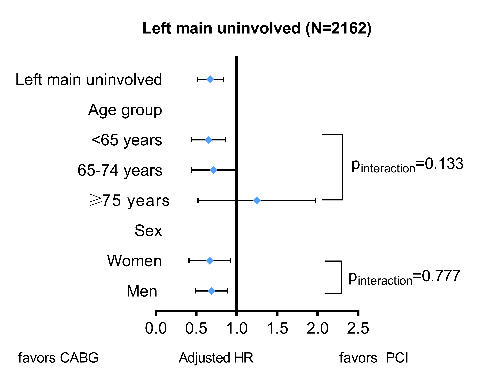

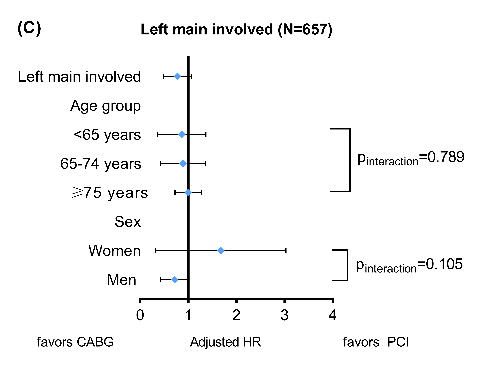

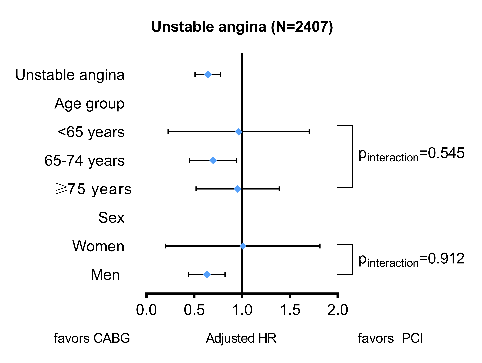

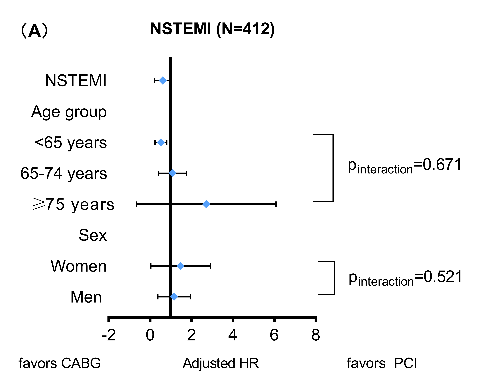

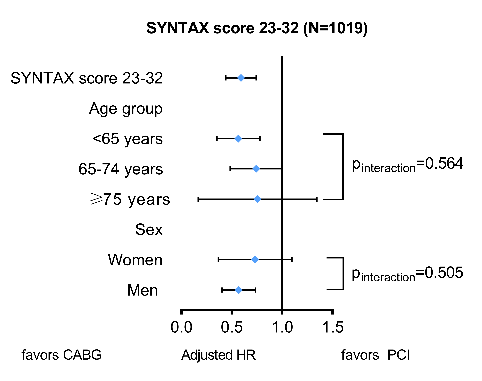

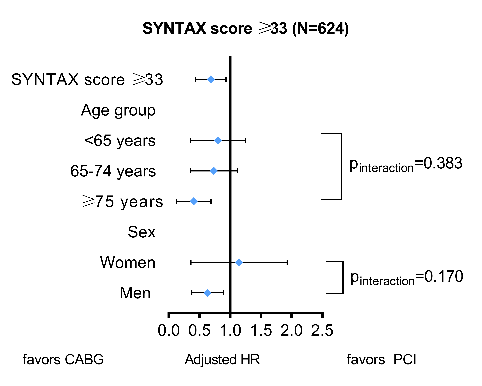

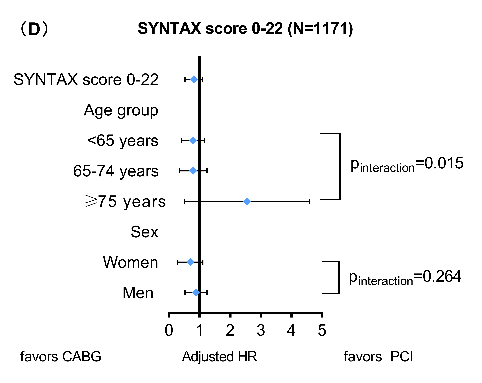


(A) NSTEMI and unstable angina population; (B) diabetes and non-diabetes population; (C) left main involved and uninvolved population; (D) different SYNTAX score groups. IPTW, inverse probability of treatment weighting; CABG, coronary artery bypass grafting; PCI, percutaneous coronary intervention; MACCE, major adverse cardiovascular and cerebrovascular event; NSTEMI, non-ST-segment elevation myocardial infarction; SYNTAX, synergy between percutaneous coronary intervention with taxus and cardiac surgery.
